# MENet: A Mitscherlich function based ensemble of CNN models to classify lung cancer using CT scans

**DOI:** 10.1371/journal.pone.0298527

**Published:** 2024-03-11

**Authors:** Surya Majumder, Nandita Gautam, Abhishek Basu, Arup Sau, Zong Woo Geem, Ram Sarkar

**Affiliations:** 1 Department of Computer Science and Engineering, Heritage Institute of Technology, Kolkata, India; 2 Department of Computer Science and Engineering, Jadavpur University, Kolkata, India; 3 Department of Computer Science and Engineering, National Institute of Technology Durgapur, Durgapur, India; 4 College of IT Convergence, Gachon University, Seongnam, South Korea; Nanjing Normal University, CHINA

## Abstract

Lung cancer is one of the leading causes of cancer-related deaths worldwide. To reduce the mortality rate, early detection and proper treatment should be ensured. Computer-aided diagnosis methods analyze different modalities of medical images to increase diagnostic precision. In this paper, we propose an ensemble model, called the Mitscherlich function-based Ensemble Network (MENet), which combines the prediction probabilities obtained from three deep learning models, namely Xception, InceptionResNetV2, and MobileNetV2, to improve the accuracy of a lung cancer prediction model. The ensemble approach is based on the Mitscherlich function, which produces a fuzzy rank to combine the outputs of the said base classifiers. The proposed method is trained and tested on the two publicly available lung cancer datasets, namely Iraq-Oncology Teaching Hospital/National Center for Cancer Diseases (IQ-OTH/NCCD) and LIDC-IDRI, both of these are computed tomography (CT) scan datasets. The obtained results in terms of some standard metrics show that the proposed method performs better than state-of-the-art methods. The codes for the proposed work are available at https://github.com/SuryaMajumder/MENet.

## Introduction

Lung cancer is cancer that starts in the lungs, usually in the cells that line the airways. It is one of the main reasons for cancer-related fatalities globally. According to the World Health Organization (WHO), there were 2.21 million new cases of lung cancer in 2020 [1], making it the second most common type of cancer in terms of new cases. Smoking, exposure to certain chemicals and pollutants, and a family history of the disease are all risk factors for lung cancer. Cancer significantly affects a person’s quality of life, as well as their physical and mental health. To reduce mortality and improve patient’s quality of life, awareness of cancer prevention, early detection and effective treatment options must be increased. The use of computer-aided detection (CAD) methods to help medical workers in the early detection of diseases like lung cancer has grown in popularity in recent years. These methods analyze medical images using machine learning algorithms. CAD systems can increase diagnostic precision and decrease the likelihood of human mistakes by automating the diagnosis process. Recent years have seen the publication of several study papers discussing the application of machine learning methods such as [2–4] for early detection of lung cancer. These techniques generally involve using a collection of the particular modalities of medical images to teach a machine learning algorithm and derive useful features that can be used for identifying the presence or absence of lung cancer, or the severity of the same. Over the last few decades, Convolutional Neural Networks (CNNs) [5] have been incredibly effective in overcoming several difficulties related to image classification and pattern recognition. Unlike conventional machine learning algorithms, CNNs do not need any feature-engineering methods, rather they can automatically extract the relevant features from the input data. CNNs, a subclass of deep neural networks, have transformed the field of computer vision by getting cutting-edge results on a variety of visual recognition tasks, including image segmentation, object detection, and classification. CNNs are a powerful tool used for different applications, but they are not a silver bullet and may require support from other methods to improve performance [6, 7].

Also, training deep neural networks from scratch requires large amounts of data and computational resources, and can take a long time to converge. In some cases, it may be impractical or impossible to form a deep network from scratch due to these limitations. Transfer learning provides a solution [8, 9] that allows us to use pre-trained models that have been trained on large datasets and learned useful feature representations. By using pre-trained models or transferring knowledge from one task to another, transfer learning can boost the performance of a single model. By lowering the variance and enhancing the models’ capacity for generalization, ensembling multiple models can further enhance performance. Ensemble learning can assist in building more reliable and precise models that are better able to manage real-world data by combining the decision-making abilities of varied models. The use of component classifiers learned from various groups to create a composite categorization system was suggested by [10] in order to improve the performance of identification systems. The popularity of ensemble learning has grown steadily in recent years [11–17]. Nowadays, ensemble learning is considered a key technique in the machine learning toolkit and is widely used in industry and the academic world. There are several approaches like bagging [18], boosting [19], and staking [20] for classification tasks in ensemble learning based on the specific application and available data.

**Fig 1:**
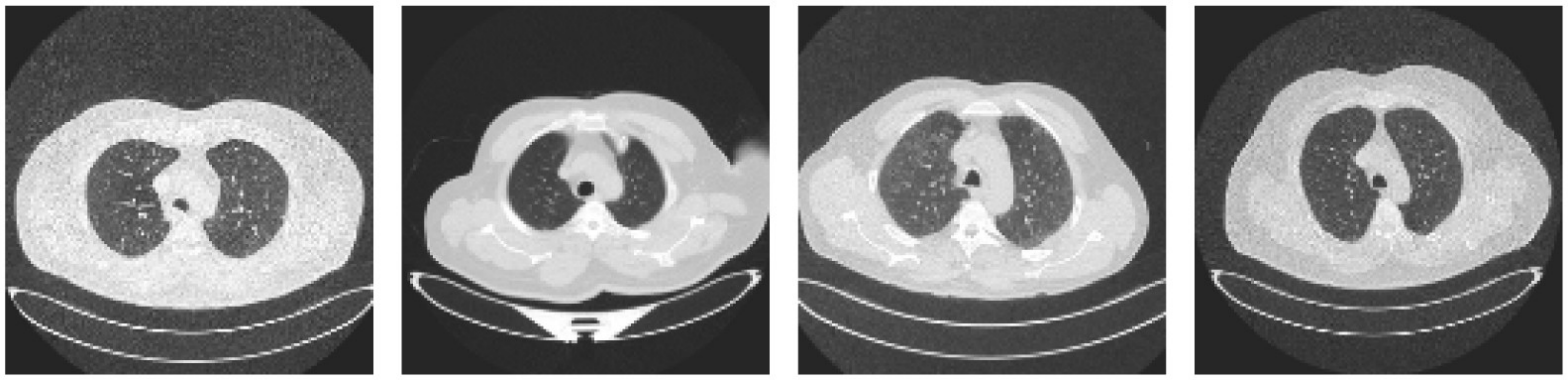
CT scan images showing noise and distortion, low-density contrast, and quality variation, respectively.

**Fig 2:**
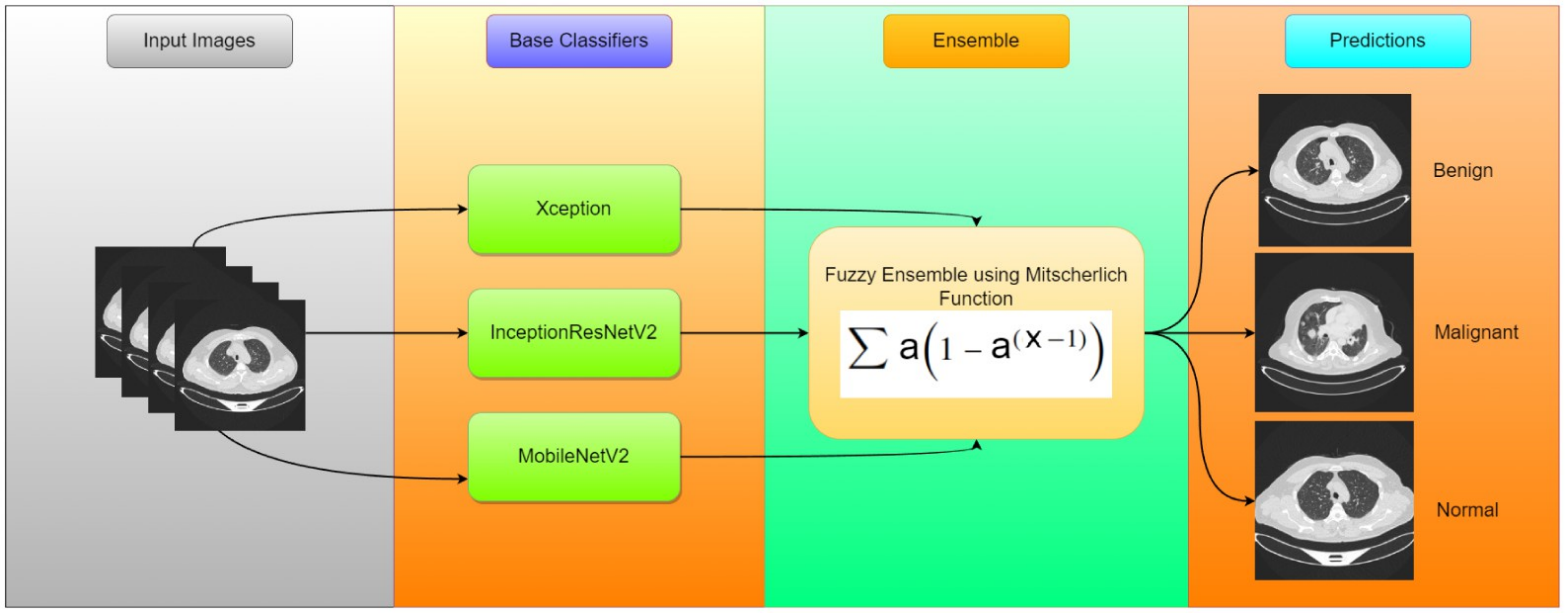
Overall pipeline of the proposed model, called MENet, highlighting the base models and the ensemble procedure.

Considering these details, in this study, we have assumed that ensemble learning can be an effective way to detect lung cancer, which involves identifying different types of lung abnormalities from medical computed tomography (CT) scans. This approach may be helpful to tackle few challenges, such as image quality variations, noise, distortion, low-density contrast, etc., as shown in Fig 1. The overall pipeline of the proposed model is represented in the diagram presented in Fig 2. The main **contributions** of this work are as follows:

- We propose an ensemble model, called MENet, for lung cancer classification using CT scans.
- MENet combines confidence scores obtained from three transfer learning-based CNN models, namely Xception, InceptionResNetV2, and MobileNetV2.
- We also propose a fuzzy ranking system based on the Mitscherlich function to rank and combine the outputs of different base classifiers for forming an ensemble-based prediction model.
- Our proposed method is trained and tested on two publicly available lung CT scan datasets, namely IQ-OTHNCCD and LIDC-IDRI.
- MENet outperforms the existing results in lung cancer prediction with an accuracy of 99.54% and 95.75% on IQ-OTHNCCD and LIDC-IDRI datasets, respectively.

## Literature review

The paper’s literature review has two distinct segments. The first segment presents a comprehensive summary of previous studies conducted on lung cancer classification. The second segment concentrates on examining ensemble techniques, specifically in the realm of medical image processing.

### A review of lung cancer classification

In the study, [21] presented an approach for the classification of lung nodules and non-nodules in CT images, which involved the utilization of texture features. They used three strategies to extract texture measurements: rose diagrams (RD), artificial crawlers (ACs), and a hybrid model that incorporates ACs & RD. The authors employed a support vector machine (SVM) classifier with a radial basis kernel to differentiate between nodules and non-nodules in the candidate categorization process. The study used the Lung Image Database Consortium (LIDC-IDRI) image database and achieved a mean specificity of 94.78%, mean sensitivity of 91.86%, and mean accuracy of 94.30%. However, the main limitation of this work is that the comparison with previous methods is only approximate due to differences in databases and test scenarios. The only precise comparison is with [22], and the proposed methodology achieved a lower specificity score, indicating its lesser ability to classify non-nodule cases accurately. Marcin et al. [23] introduced a novel approach to classify lung carcinomas. This method involves the localization and extraction of lung nodules by computing the local variance of pixels, detecting the maxima, and utilizing a probabilistic neural network as a classifier. The simplicity of the proposed method enables it to detect low-contrast nodules and reduce computational workload while maintaining performance. To explain the nodule appearance without ignoring spatial information, a 7th Order Markov Gibbs Random Field and a Local Binary Pattern are devised by Shaffie et al. [24].

Netto et al. [25] proposed a methodology for analyzing lung lesions using temporal evaluation, which can aid in the diagnosis of indeterminate lesions during treatment. The modified quality threshold clustering technique was employed to assign each voxel of the lesion to a cluster, and the alteration in the lesion was evaluated by analyzing the movement of voxels to other clusters over time. To differentiate between benign and malignant lesions, statistical features were extracted. The authors employed two databases of pulmonary lesions, one for malignant lesions under treatment and the other for undetermined cases, to develop their proposed methodology. By analyzing the density changes of lesions over time, the researchers achieved an accuracy of 98.41% in accurately identifying lung lesions. Xie et al. [26] proposed a distinctive approach for lung nodule classification, named Fuse-TSD. This method combines information on texture, shape, and deep model-based learning at the decision level. The approach utilizes a texture descriptor derived from a gray-level co-occurrence matrix (GLCM), a Fourier shape descriptor that captures the heterogeneity of nodules, and a deep convolutional neural network (DCNN) that learns the feature representation of nodules automatically, slice-by-slice. The obtained characteristics are trained with an AdaBoosted back propagation neural network (BPNN), and the judgments of three classifiers are combined to distinguish benign from malignant nodules.

An automated diagnosis classification method for CT lung images was introduced by [27]. The method employs an Optimal Deep Neural Network and Linear Discriminate Analysis to extract deep features and reduce dimensionality. To optimize the Optimal Deep Neural Network for classifying lung nodules as benign or malignant, the authors used a modified gravitational search algorithm. The automated approach not only enhances the classification accuracy but also reduces the time required for manual labeling and prevents human errors in recognizing normal and abnormal lung images. Shafi et al. [28] proposes deep learning supported SVM model for CT images and gain accuracy of 94%. A lightweight, multi-view sampling-based multi-section CNN architecture was introduced by [29], which efficiently captures the structural information of nodules from CT scans for lung cancer diagnosis. The method utilizes a view pooling layer to aggregate information from multiple cross-sections of the nodule. It encodes its volumetric information into a concise representation that is then utilized for nodule classification. Masood et al. [30] developed a decision support system using CT scans for lung nodule detection. Their approach utilized a 3D deep CNN, multi-region proposal network, and median intensity projection to automatically identify regions of interest. The performance of the system was evaluated on LUNA16, ANODE09, and LIDC-IDRI datasets. The main shortcoming of this work is that the accuracy of detecting micro-nodules with a diameter of less than 3 mm is relatively low.

Lin et al. [31] put forth a framework for classifying lung cancer using Generative Adversarial Networks (GANs) and discriminator networks. They utilized a deconvolution layer, leaky Rectified Linear Unit (ReLU) activation function, and batch normalization to obtain a 64×64×3 image. The main objective of their research was to overcome the challenge of sparse image data and generate precise predictions. Zhao et al. [32] proposed a forward and backward GAN with multi-scale VGG16. Yuan et al. [33] proposed a CAD method to enhance the detection of pulmonary nodules on CT scans. Their proposed method utilized a 3D Residual U-Net model along with a multi-branch classification network to achieve a high detection sensitivity of 94.0% and a competition performance metric (CPM) score of 0.959 on the LUNA 2016 dataset through multi-task learning. Bhatia et al. [34] obtained an accuracy of 84% using the same model on the LIDC-IDRI dataset. Halder et al. [35] presented an end-to-end system for detecting and classifying lung nodules from high-resolution CT images using atrous convolution. They achieved high-performance indices, with the proposed architecture, ATCNN2PR, consisting of a two-layer atrous pyramid and residual connections, demonstrating the highest classification accuracy. The results showed that the system outperformed other competing frameworks with an accuracy, specificity, and sensitivity of 95.97%, 96.89%, and, 95.84%, respectively.

### Survey of ensemble techniques

Ensemble learning is a technique that combines many learning models to increase the overall accuracy and resilience of the learning framework. In this section, we will give a quick overview of various recently proposed ensemble learning approaches with an emphasis on medical picture analysis. Maji et al. [36] introduced a computational imaging system for the detection of blood vessels in fundus color images using ensemble learning and deep learning. Their approach involved training an ensemble of deep CNNs to segment vessel and non-vessel regions of a color fundus image. During inference, the responses of individual ConvNets within the ensemble were combined by averaging to generate the final segmentation. The method was evaluated using the DRIVE database, and the results showed a maximum average accuracy of 94.7%. Kassan et al. [37] presented an ensemble deep learning-based approach for binary classification of breast histopathology images. They utilized three pre-trained CNNs for feature extraction and a multi-layer perceptron classifier for the final classification. The proposed technique outperformed individual classifiers and other machine learning algorithms in terms of prediction accuracy on four benchmark datasets. Bhowal et al. [38] developed a CAD system for breast cancer detection using a Choquet Integral fusion technique that considers subsets of classifiers. For the categorization of breast cancer histology images, their proposed method included InceptionV3, Xception, VGG19, VGG16, and InceptionResNetV2. To address the complexity of calculating fuzzy measurements, the authors used the Coalition Game, Information Theory, and constructed a novel mathematical function. The ICIAR 2018 Grand Challenge on Breast Cancer Histology (BACH) photos were utilized for evaluation, which included 2-class and 4-class tasks. The fusion method outperformed all individual models with a test accuracy of 96% for the two-class problem. Similarly, the fusion approach achieved a test accuracy of 95% for the four-class problem. Table 1 shows some recent applications of ensemble learning in the field of medical imaging.

**Table 1:** Applications of ensemble learning in medical image analysis.

Application Domain	Reference
Glioma detection	[39]
COVID-19 detection	[40–43]
Breast cancer	[44–48]
Colorectal polyp classification	[49]
Brain cancer detection	[50]
Lymphoblastic leukemia cell image analysis	[51]
Alzheimer’s disease classification	[52]
Monkeypox detection	[53]

In our proposed ensemble approach, we use the Iraq-Oncology Teaching Hospital/National Center for Cancer Diseases (IQ-OTH/NCCD) [54] lung cancer dataset taken over a period of three months (in the fall of 2019 and published in 2020). The dataset contains CT scan images of healthy and lung cancer patients who were diagnosed through different stages of lung cancer. This dataset is publicly available [55–57] and contains three classes of images. Among the three classes, the benign class contains 120 images, the malignant class contains 561 images, and the other 461 images from normal patients as shown in Table 2. We took 70% of the data to train the models, 20% data for validation, and the remaining 10% for testing.

**Table 2:** Distribution of images found in the IQ-OTHNCCD dataset into Benign, Malignant, and Normal classes.

Dataset	Class	No. of images
IQ-OTHNCCD	Benign	120
	Malignant	561
	Normal	461

## Proposed methodology

The suggested ensemble-based lung cancer classification model (MENet) is described in-depth in this section. In order to determine the correct class of the test data, we first provide a brief description of every base learner that produces scores of confidence for an incoming lung image. These scores are then further fused via the suggested ensemble methodology.

### Deep neural based classifiers

CNNs are preferred over other types of machine learning algorithms for image classification tasks, including the detection of lung cancer from medical imaging datasets. One of the main reasons for this is that CNNs are specifically designed to handle spatial data such as images. They can learn features from the input data by using convolutional layers and pooling layers combination, which can effectively capture local patterns and spatial relationships between pixels. This makes them well-suited for analyzing medical images, which often contain complex structures and patterns. Additionally, CNNs can automatically learn and adapt to the features of the input data, without the need for manual feature engineering. Overall, these characteristics make CNNs a powerful tool for accurately classifying medical images, including those of lung cancer and have the potential to increase diagnosis precision and speed. Hereafter an exhaustive set of experiments, we have successfully settled down with Xception, InceptionResNetV2, and MobileNetV2 models. The choice of these architectures likely balances a trade-off between accuracy and computational efficiency.

#### Xception

**Fig 3:**
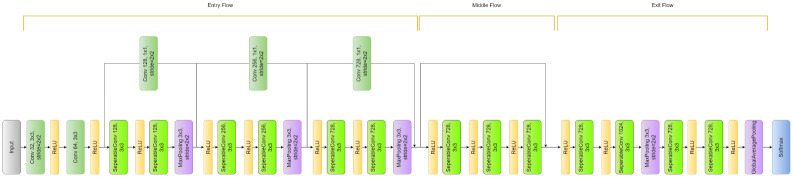
Architecture of the Xception model.

Xception is a convolutional neural network architecture proposed by [58]. It is an extension of the Inception architecture and is named “Extreme Inception” because it uses depthwise separable convolutions instead of standard convolutions used in Inception. The depth-wise separable convolution layer blocks make up the bulk of the Xception architecture, which is then followed by batch normalization and ReLU activation. The depthwise separable convolution layers are made up of two distinct layers: a pointwise convolution layer that applies a 1x1 convolutional filter to combine the output channels of the depthwise convolution and a depthwise convolution layer that applies a single convolutional filter to each input channel. A global average pooling layer and a fully connected layer for classification, constitute the final layer of the network. The depthwise separable convolutions are more computationally efficient than conventional convolutions and aid in lowering overfitting, and the residual connections enable deeper network topologies while addressing the vanishing gradient issue. This makes Xception a powerful and effective model for image classification tasks. The Xception architecture is illustrated in Fig 3.

#### InceptionResNetV2

**Fig 4:**
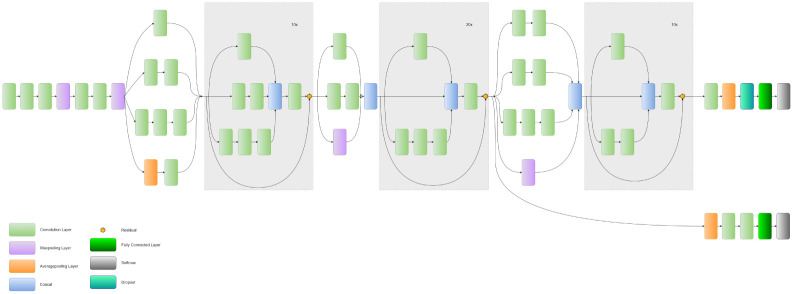
Architecture of the InceptionResNetV2 model.

Introduced by [59], InceptionResNetV2 combines the strengths of Inception and ResNet architectures. It utilizes residual connections with Inception modules, which not only address the vanishing gradient problem but also enable more complex network designs. Additionally, the model requires fewer parameters to achieve high accuracy and can quickly learn and extract features from input data. Overall, the InceptionResNetV2 architecture is a powerful tool for image classification tasks, offering both high accuracy and efficient computation. The InceptionResNetV2 architecture is shown in Fig 4.

#### MobileNetV2

**Fig 5:**
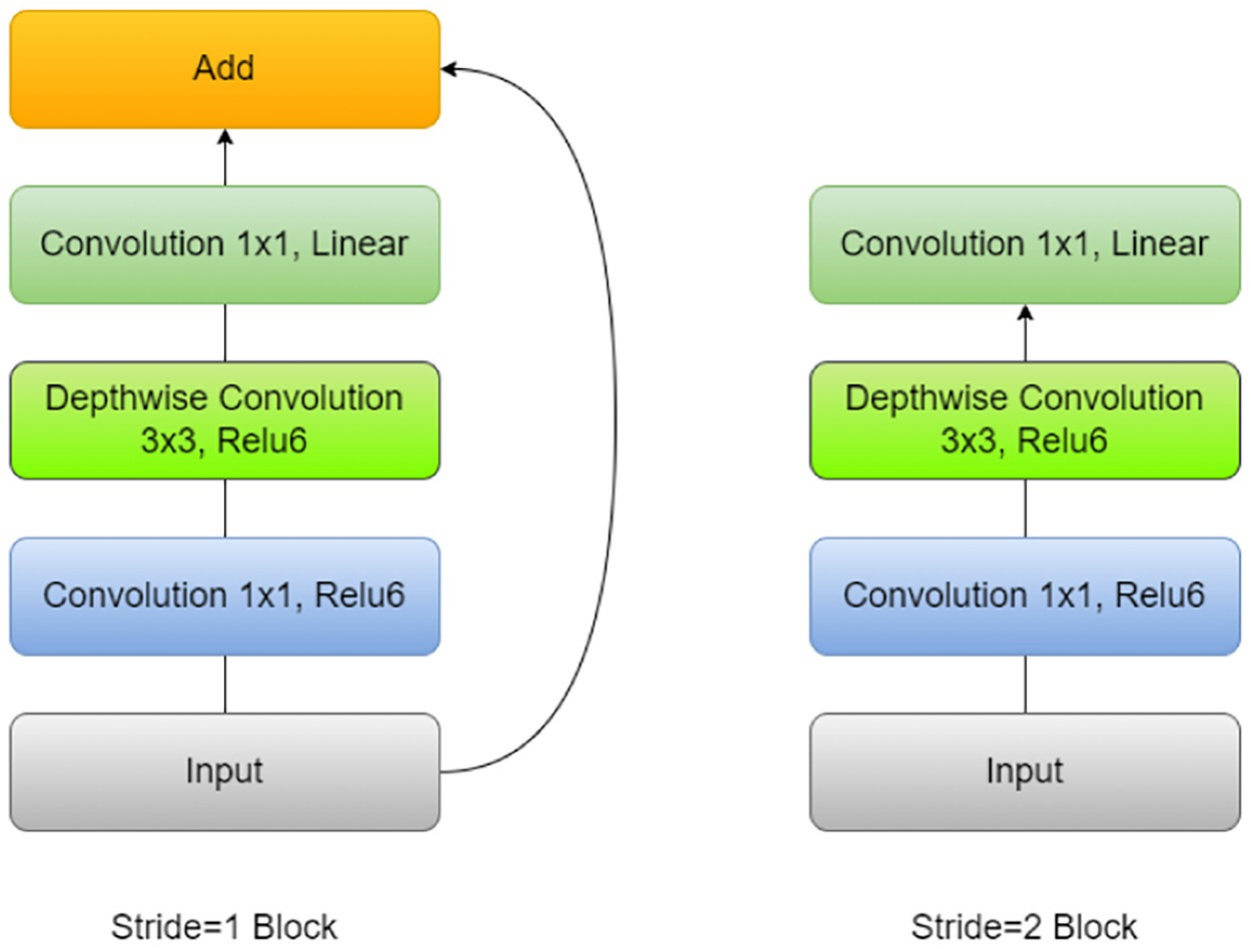
Architecture of the MobileNetV2 model.

MobileNetV2, proposed by [60] is a convolutional neural network designed for mobile and embedded vision applications. It uses depthwise separable convolution, inverted residuals, and linear bottlenecks to improve efficiency while preserving representational power. It includes two types of blocks: residual blocks with a stride of 1 and blocks with a stride of 2 for downsizing. Both types of blocks consist of three layers. The first layer in each block is a 1×1 convolution with ReLU6 activation. The second layer is a depthwise convolution, which applies a separate convolutional filter to each input channel. The final layer in each block is another 1×1 convolution but without any non-linearity. A width multiplier is used in this network to optimize the network for different hardware and resource constraints. MobileNetV2 is a highly efficient and lightweight model, making it suitable for deployment on mobile devices and other resource-constrained environments. Despite its small size, MobileNetV2 achieves high accuracy on a wide range of vision tasks, including image classification, object detection, and semantic segmentation. The architecture of MobileNet is shown in Fig 5.

### Proposed fuzzy-ensemble method

The main goal of our proposed approach is to provide greater adaptability and freedom in handling datasets with varying degrees of complexity. Using a fuzzy ranking-based approach, we can consider the uncertainty of each classifier’s predictions and assign different levels of importance to each classifier based on its performance on a particular test case. In the proposed methodology, the three CNN-based classifiers (Xception, InceptionResNetV2, and MobileNetV2) are used to detect lung cancer cases from CT scans, and fuzzy ranks are generated for each of them using the re-parameterized Mitscherlich function. To increase the overall accuracy of the classification, these fuzzy ranks are then combined using an ensemble method. The Mitscherlich function, which has been applied to the field of machine learning by [61, 62], is based on the idea of crop yield response [63] to various levels of fertilizer application [64]. Here, it is used to combine the outputs of different models with various strengths and weaknesses.

### Significance of Mitscherlich function

The Mitscherlich function plays a pivotal role in our rank-based fuzzy ensemble logic. Its selection was made based on a multitude of factors, which we shall delve into, in this section. The Mitscherlich function serves as a crucial tool for illustrating the performance of each model within our ensemble as a function of input strength. The function has a similarity to linear mapping within the (0,1) range. To address this concern, we will provide a more comprehensive explanation of our rationale and support it with relevant discussions and experiments.

In essence, the Mitscherlich function allows us to create a dose-response curve that portrays each model’s performance concerning the input strength. This curve represents the relationship between the weighted sum of the models’ predictions and the actual outcomes, yielding a value that quantifies the fit between predictions and actual results. Here’s where its unique value becomes apparent: we employ this function to generate fuzzy ranks for each class from the ensemble’s individual models. The process involves ranking the confidence scores of each class provided by the base classifiers. These fuzzy ranks are then employed in ensemble predictions on a validation set, specifically within the top K ranks. Why is this important? Well, it enables us to construct an adaptable ensemble model capable of dynamically adjusting the ranking of individual models based on the specific characteristics of each input instance. As the decision score for a class, accurately predicted by a classifier, approaches one, the Mitscherlich function exhibits a steeply dropping nature within the domain range of 0 to 1. This characteristic proves highly advantageous when forming an ensemble of decision scores from various learning models. It ensures that as we approach a confident prediction (score near one), the Mitscherlich function’s response is highly sensitive, effectively distinguishing between models with slight performance variations in this critical region.

### Implementation of the ensemble model

There should be M different decision scores (also known as confidence scores of classifiers) such as *CoF*^(1)^, *CoF*^(2)^, …, *CoF*^(*M*)^ for each input image P. As we utilized three different CNN-based models to produce the scores of confidence on the dataset, M, in our case, equals 3. In the Eq 1, the decision scores from the dataset are normalized, where C is the total count of classes in the dataset under consideration.

$$\begin{array}{c} \sum_{c=1}^{C}CoF_{c}^{\left(i\right)}=1;\forall i,\;i=1,2,3,\ldots ,M \end{array}$$ (1)

Fuzzy ranks are generated using the scores of confidence of each sample in the dataset, which are divided into three different classes. The Mitscherlich function produces the fuzzy rank for a class c using the *i*_*th*_ classifier’s scores of confidence as shown Eq 2.

$$\begin{array}{c} R_{c}^{\left(i\right)}=2\left(1-2^{CoF_{c}^{\left(i\right)}-1}\right);\forall i,c,\;i=1,2,3,\ldots ,M;c=1,2,\ldots ,C \end{array}$$ (2)

**Fig 6:**
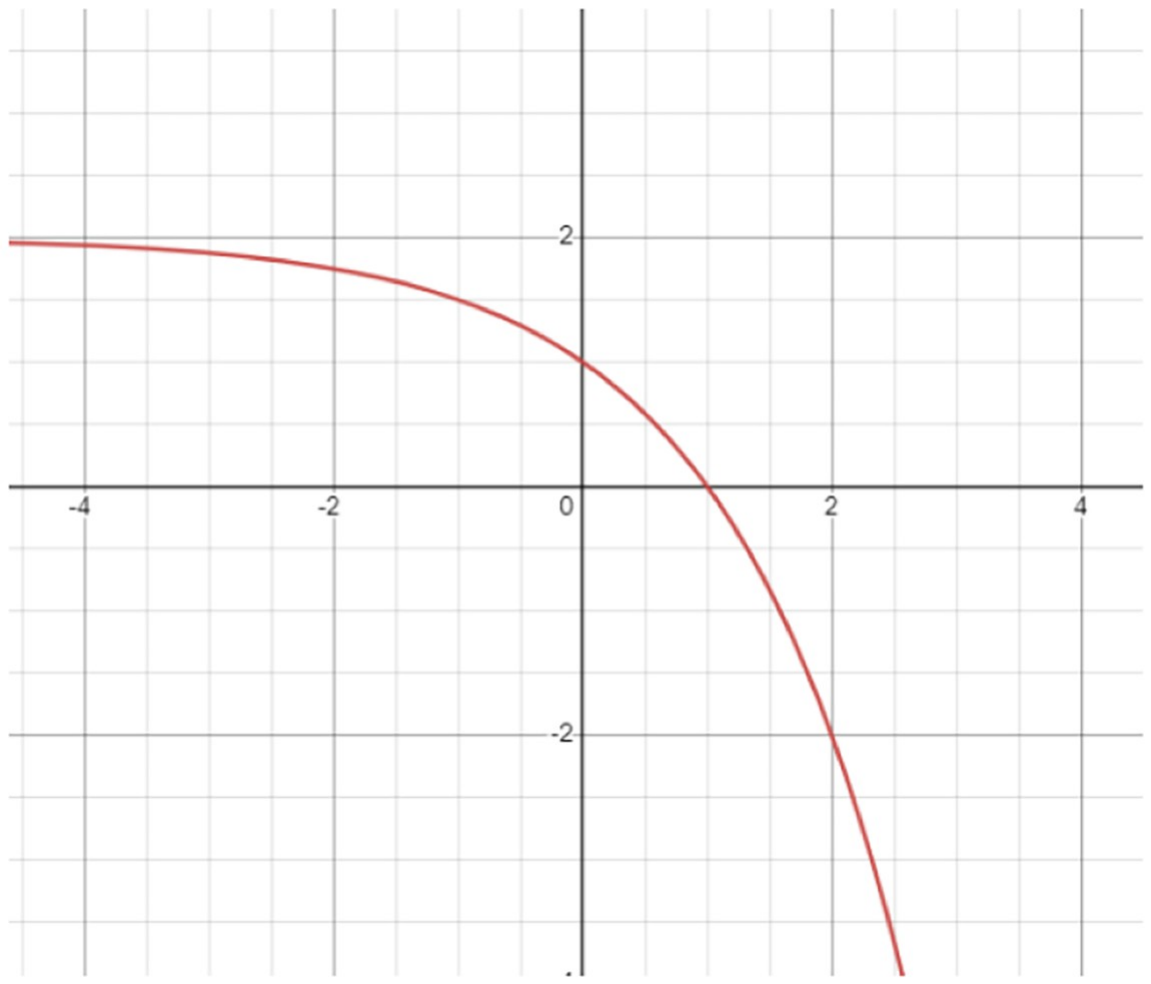
Graphical representation of the modified Mitscherlich function used in the present work.

The Fig 6 shows a pictorial depiction of the modified Mitscherlich function graph, as mentioned in Eq 2. The value of $R_{c}^{\left(i\right)}$ is in-between 0 and 1, with the lowest value 0 being equivalent to rank 1 (best rank), i.e., a greater score of confidence results in a lower (better) value of rank. The fuzzy rank sum (*FRS*_*c*_) and complement of the confidence factor sum (*CCFS*_*c*_) are computed as follows if *K*^(*i*)^ is used to represent only the top k ranks, that is, rank 1, 2,…, k, that corresponds to class c are as follows:

$$\begin{array}{c} FRS_{c}=\sum_{i=1}^{M}\{\begin{array}{cc} R_{c}^{\left(i\right)}, & \text{if}\;R_{c}^{\left(i\right)}\in K^{\left(i\right)}\\ P_{c}^{R}, & \text{otherwise} \end{array} \end{array}$$ (3)

$$\begin{array}{c} CCFS_{c}=\frac{1}{M}\sum_{i=1}^{M}\{\begin{array}{cc} CoF_{c}^{\left(i\right)}, & \text{if}\;R_{c}^{\left(i\right)}\in K^{\left(i\right)}\\ P_{c}^{CoF}, & \text{otherwise} \end{array} \end{array}$$ (4)

If class *c* does not fall among the top k class ranks, penalties $P_{c}^{R}$ and $P_{c}^{CoF}$ are applied to it. Using the aforementioned Mitscherlich function, the value of $P_{c}^{R}$ is set to 1, which is determined by setting $CoF_{c}^{\left(i\right)}=0$; and the value of $P_{c}^{CoF}$ is set to 0. These penalty values prevent class *c* from emerging as an improbable winner. The combination of *FRS*_*c*_ and *CCFS*_*c*_, which is used to produce the ensemble model’s final predictions, yields the decision score in question. Eq 5 determines the final decision score (*FDS*).

$$\begin{array}{c} FDS_{c}=FRS_{c}\;*\;CCFS_{c} \end{array}$$ (5)

Finding the class with the lowest *FDS* value yields the final projected class for data instance **I**, which is provided as shown in Eq 6.

$$\begin{array}{c} class\left(I\right)=\underset{c=1,2,\ldots ,C}{\text{arg}\;\text{min}}\;FDS_{c} \end{array}$$ (6)

A dry run of the proposed fuzzy rank-based ensemble method is shown under S1 Appendix.

## Results and discussion

In this section, we report the detailed results and the corresponding analysis of the proposed ensemble of CNN models used for lung cancer detection in CT scans. The distribution of images in the dataset is already provided in Table 2. The implications of the obtained results are also discussed. In addition, we present a comparative evaluation to show that the proposed method outperforms other models and commonly used ensemble techniques implemented in the literature.

### System configuration

The entire set of experiments has been run in Jupyter equipped with a 12 GB NVIDIA Tesla T4 GPU that has been made available by Google Colab. Python 3 environment, along with open-source modules such as Tensorflow, Keras, Matplotlib, Scikit, Numpy, and Pandas is utilized for the implementation.

### Evaluation metrics

Evaluation metrics are important in assessing the effectiveness and strength of a predictive/learning model. It is important to use a variety of standard evaluation metrics to get a complete picture of the model’s performance and to ensure that it meets the requirements of the problem under consideration. In classification problems, these metrics are used to evaluate the performance in predicting the correct class label for a given input. Table 3 shows various performance measures used in our classification method. Consider there is a two-class classification problem, where one class is termed as ‘positive’ and another one is termed as ‘negative’. Most of these measures are computed using a confusion matrix employing four fundamental components, such as true positive (*T*^*P*^) rate, true negative (*T*^*N*^) rate, false positive(*F*^*P*^) rate, and false negative(*F*^*N*^) rate.

**Table 3:** Evaluation matrix used in our classification methods.

Metric	Formula
Accuracy(Acc)	$\frac{T^{P}+T^{N}}{T^{P}+T^{N}+F^{P}+F^{N}}$
Precision(Pre)	$\frac{T^{P}}{T^{P}+F^{P}}$
Recall(Re)	$\frac{T^{P}}{T^{P}+F^{N}}$
F1-Score(F1)	$\frac{T^{P}}{T^{P}+\frac{1}{2}\left(F^{P}+F^{N}\right)}$

### Implementation

Initially, we perform extensive experimentation with different combinations of CNN models to determine the best combination of base learners for our proposed ensemble technique. The hyperparameters selected for this experiment are mentioned in Table 4. The results of the experimentation are shown in Table 5.

**Table 4:** Hyperparameters of the base classifiers.

Hyperparameter	Value/Name
Optimizer	Adam
Loss function	Sparse Categorical Cross Entropy
Learning rate	0.001
No. of epochs	60

**Table 5:** Results of experiments implemented to determine the base classifiers for forming the ensemble in this study.

Model 1	Model 2	Model 3	Accuracy (%)
NasNetMobile	MobileNet	ResNet-152	95.98
NasNetMobile	MobileNetV2	ResNet-50	94.87
NasNetMobile	MobileNetV2	DenseNet-169	95.21
NasNetMobile	MobileNetV2	InceptionResNetV2	92.56
MobileNetV2	ResNet-152	DenseNet-169	96.41
MobileNetV2	ResNet-50	DenseNet-169	92.99
MobileNetV2	ResNet-50	InceptionResNetV2	97.17
MobileNetV2	ResNet-152	InceptionResNetV2	96.67
NasNetMobile	ResNet-152	DenseNet-169	97.00
NasNetMobile	ResNet-152	InceptionResNetV2	96.41
NasNetMobile	ResNet-50	DenseNet-169	96.06
NasNetMobile	ResNet-50	InceptionResNetV2	96.92
Xception	ResNet-152	DenseNet-121	97.77
Xception	ResNet-152	InceptionResNetV2	95.04
Xception	ResNet-18	InceptionResNetV2	98.20
Xception	MobileNet	DenseNet-121	98.29
Xception	ResNet-18	MobileNetV2	98.54
Xception	MobileNet	NasNetMobile	98.12
**Xception**	**InceptionResNetV2**	**MobileNetV2**	99.54

The best accuracy is obtained by selecting Xception, InceptionResnetV2, and MobileNetV2 as the base classifers for the ensemble approach. The metric scores given by these models along with some additional information is shown in Table 6. These three models, i.e., Xception, InceptionResNetV2, and MobileNetV2 give us an accuracy score of 99.02%, 97.26%, and 99.45%, respectively. The results show that these models are very reliable for being chosen for this fuzzy ensemble.

**Table 6:** Performance measure of each model along with their total number of parameters.

Model	No. of parameters	Acc (%)	Pre (%)	Re (%)
Xception	20,867,627	99.02	99.38	99.59
InceptionResNetV2	54,341,347	97.26	88.33	93.80
MobileNetV2	2,261,827	99.45	99.59	99.79
**Proposed Ensemble**	**77,470,801**	**99.54**	**99.62**	**98.61**

**Fig 7:**
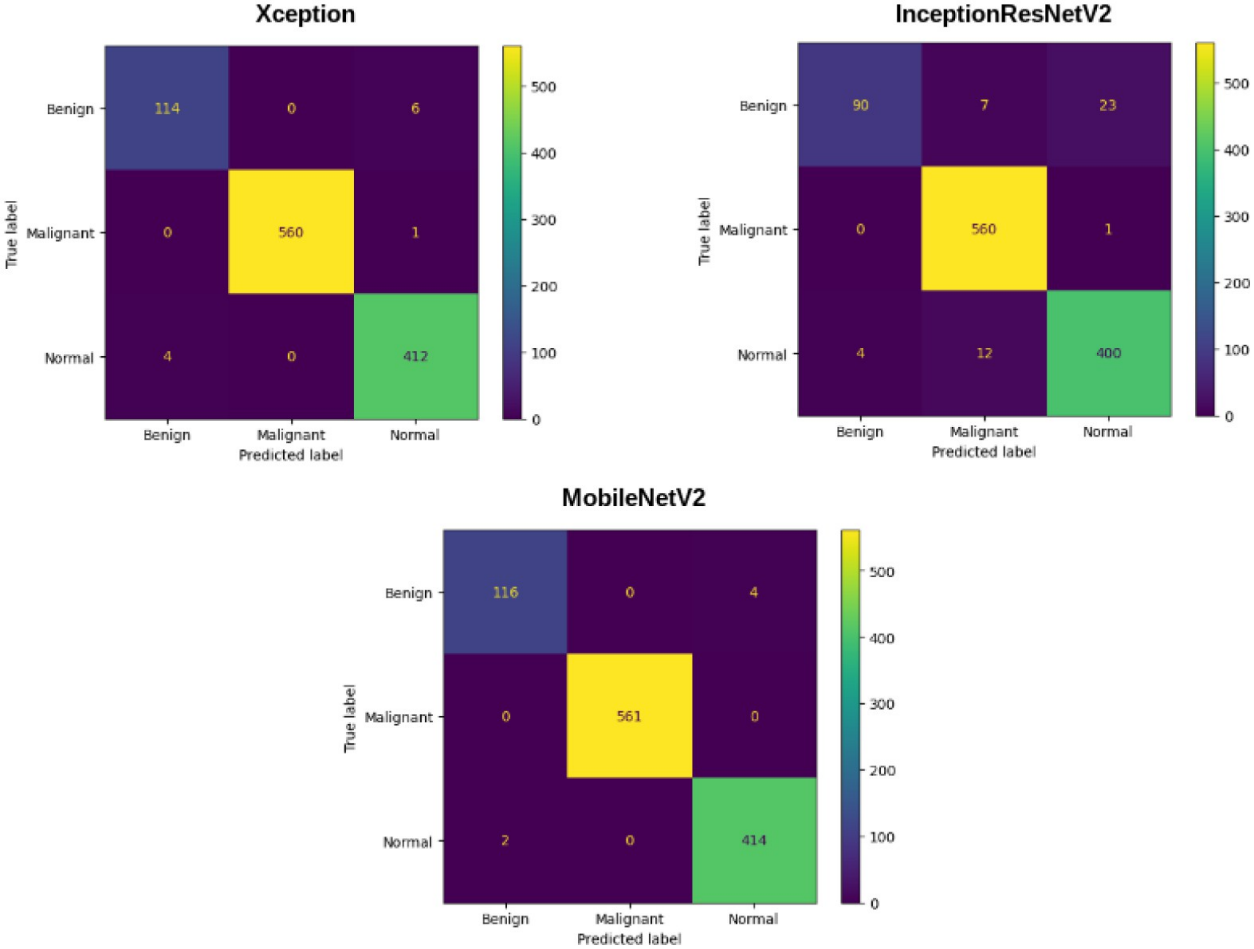
Confusion matrix obtained on the IQ-OTHNCCD dataset by Xception, InceptionResNetV2, and MobileNetV2 respectively.

**Fig 8:**
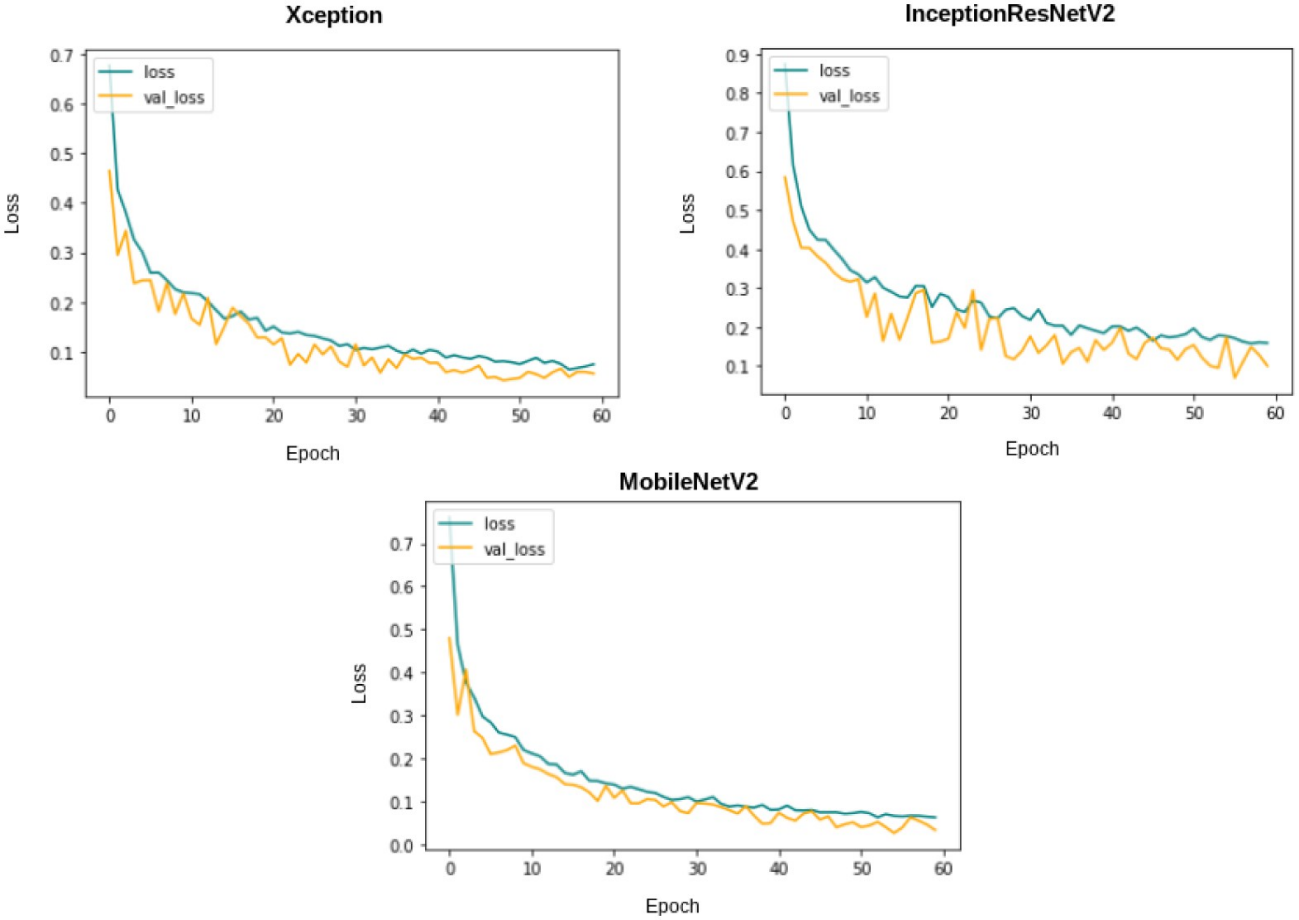
Training and validation loss plots for 60 epochs on the IQ-OTHNCCD dataset.

**Fig 9:**
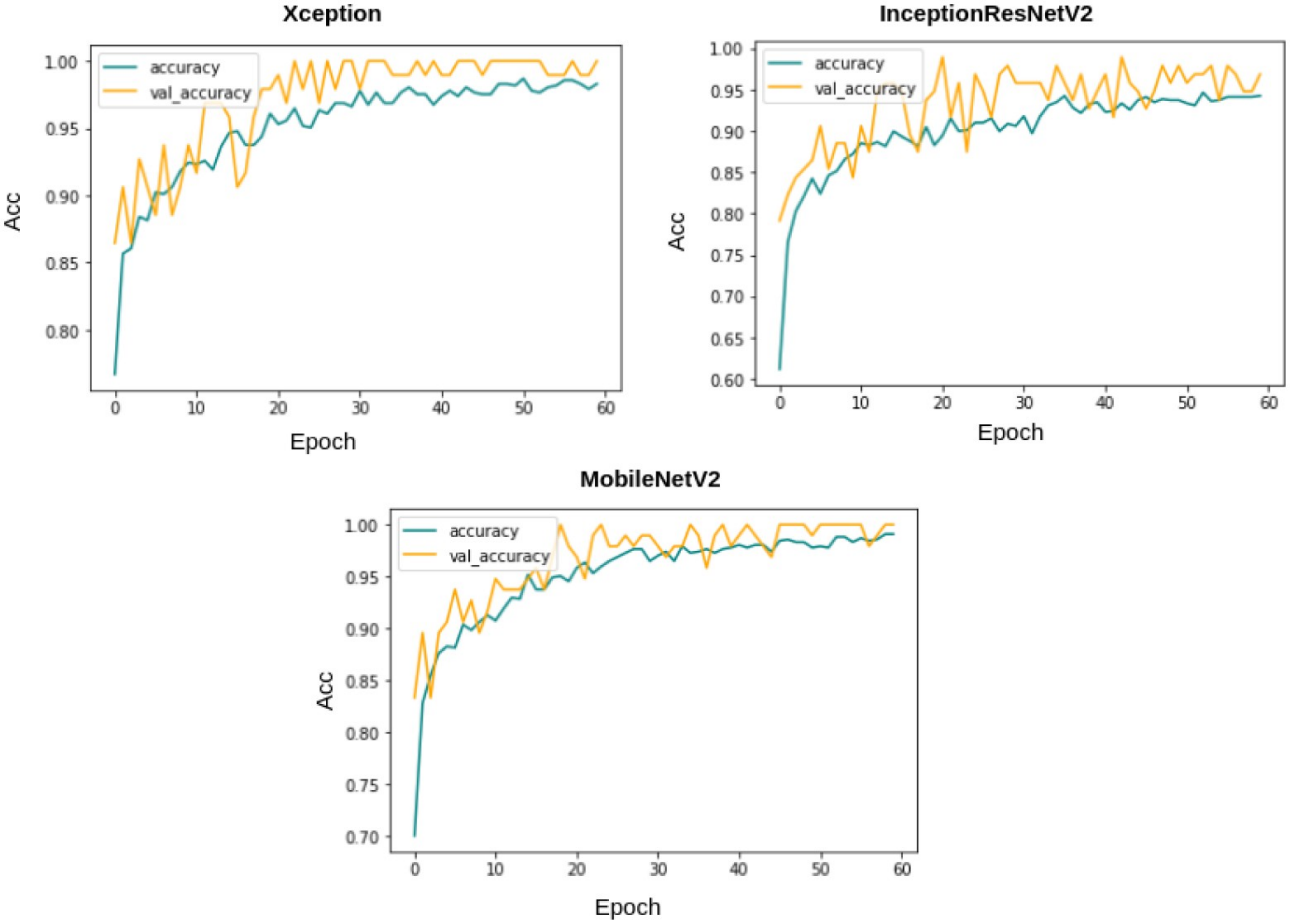
Training and validation accuracy plots for 60 epochs on the IQ-OTHNCCD dataset.

Each of these base models generates the confidence scores for each class for every single image in the dataset. These confidence scores of all the base models for each image are generated and stored for each individual classifier. For the results obtained in Table 6, the three transfer-learned models have been trained for 60 epochs with the Adam optimizer individually. Fig 7 shows their respective confusion matrices on the dataset used. Although there are some misclassifications, the ratio of misclassifications to that of the correct classifications is very low, indicating that these models are reliable for the work. The loss curves obtained by each of the base learners(or classifiers) are shown in Fig 8. As our models are all transfer learning-based models that have been pre-trained on the ImageNet dataset, we just had to fine-tune the models on our IQ-OTHNCCD lung cancer dataset. From the loss curves of the three models, we can see that there is hardly any problem with overfitting in the three models. The accuracy curves as shown in Fig 9 display the corresponding accuracy scores as shown in Table 6. After fusing the confidence scores of the three models using the proposed Mitscherlich function-based ensemble model, we get the results.

**Fig 10:**
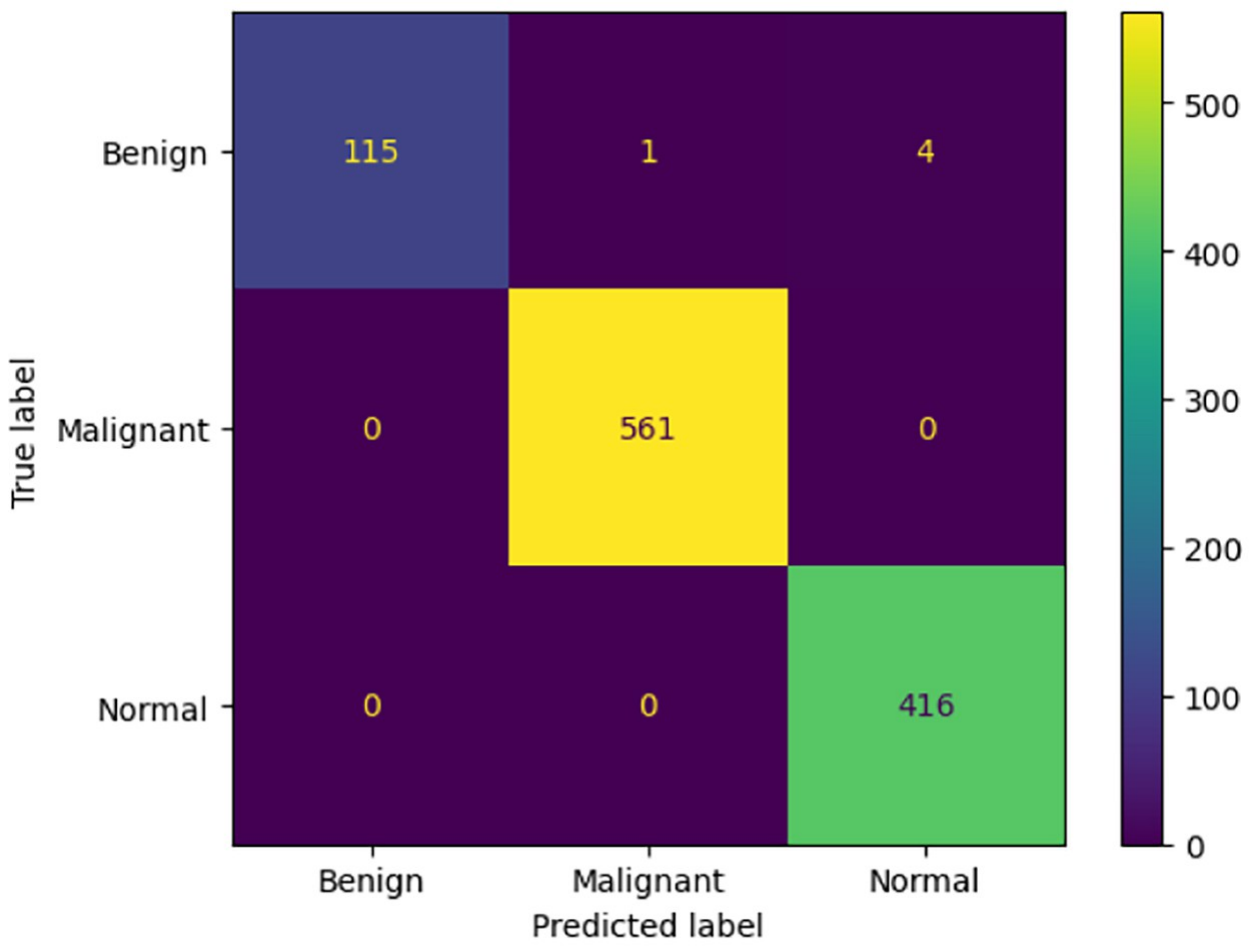
Confusion matrix of proposed ensemble using Mitscherlich function (MENet).

**Fig 11:**
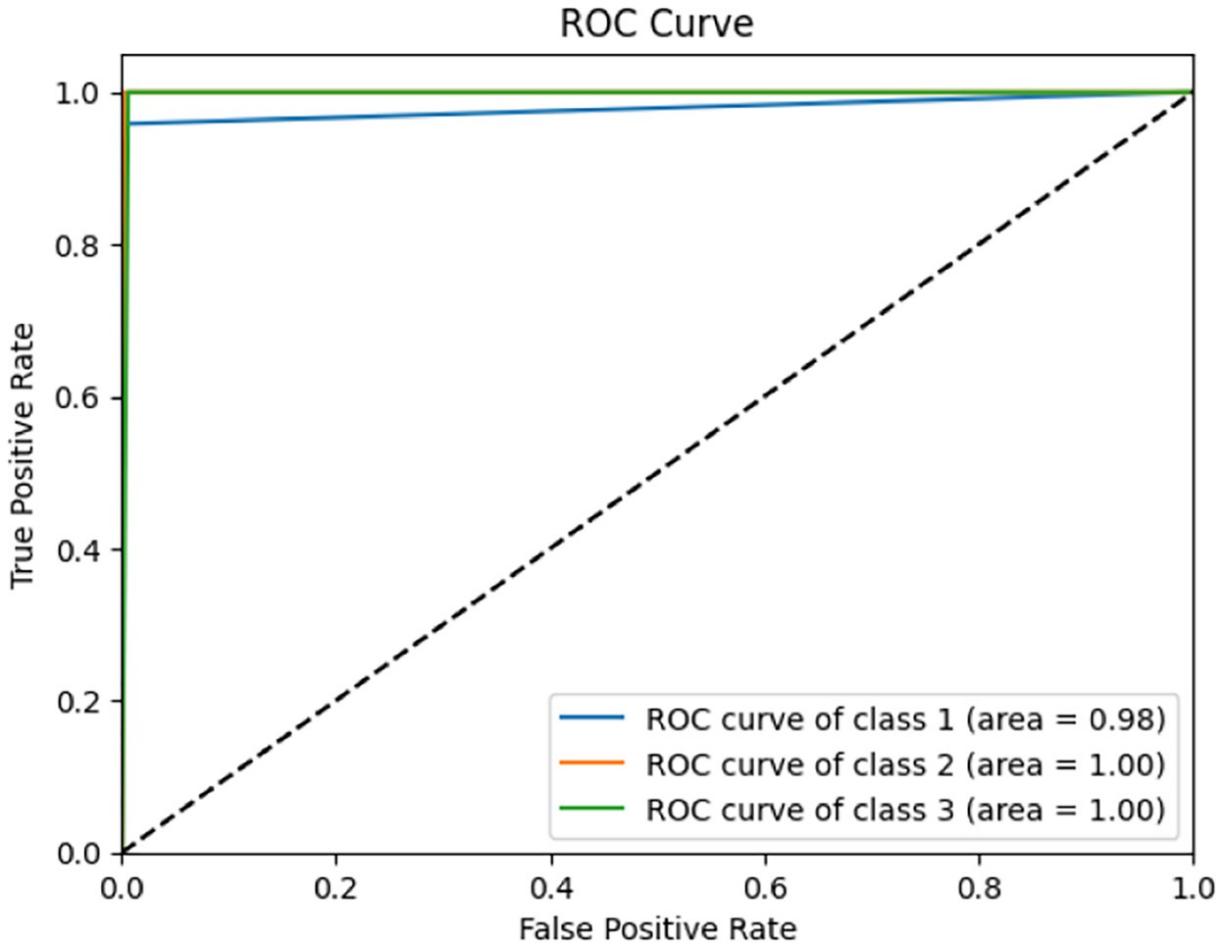
ROC-AUC curve obtained by the proposed ensemble model (MENet).

**Fig 12:**
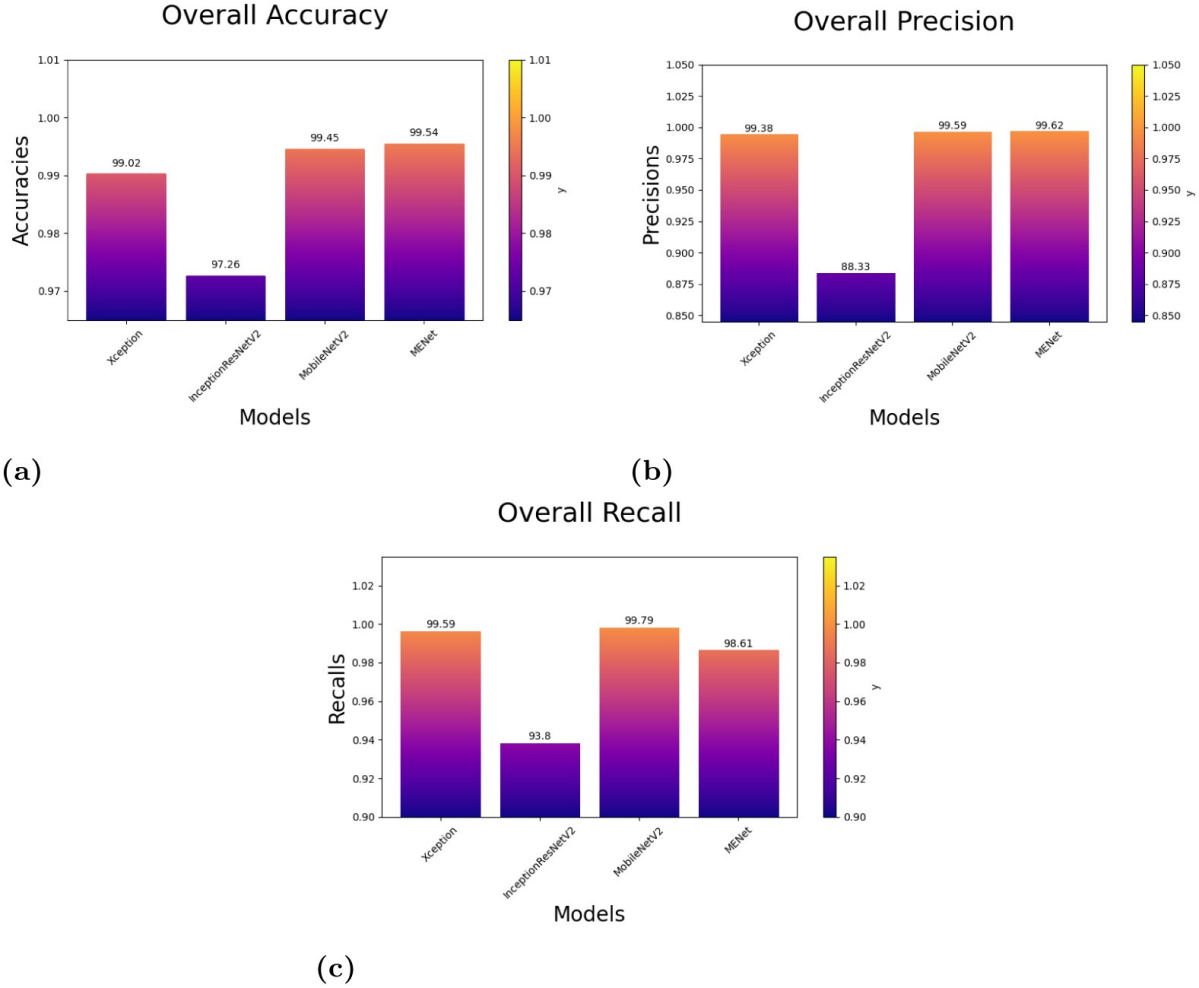
Results of the Models depicting their overall accuracies, precisions and recalls on **the IQ-OTHNCCD dataset**: (a) Overall Accuracies of the models, (b) Overall Precisions of the models, (c) Overall Recalls of the models.

From Figs 10 and 11 and Table 7, we can observe that the overall accuracy achieved is 99.54% after combining the results of the three base classifiers. The class-wise results have also been given in the Table 7. All these high classification accuracies indicate that the model is highly reliable. The final ROC curve obtained on this dataset shows that the False Positive rate is significantly low. This indicates that this proposed fuzzy ensemble method is very efficient for the problem under consideration. The Confusion Matrix of this proposed fuzzy ensemble method, as shown in Fig 10 also shows very few mispredictions than all the three base classifiers. The area under the curve (AUC) is shown in Fig 11. Here, we can observe for class 1, i.e., Benign: 98%, for class 2, i.e., Malignant: 100%, and for class 3, i.e., Normal: 100%. As the graph in Fig 11 is shifted to the top left corner, it indicates the highly efficient ability of the final model to classify test images into one of the three classes. Fig 12 provides a detailed pictorial representation of the performances of each base model and the final ensemble method.

**Table 7:** Class-wise and overall results obtained by the proposed ensemble model.

Class	Pre	Re	F1	Support	Acc	Overall Acc	Overall Re	Overall F1
Benign	1.0000	0.9583	0.9787	120	0.95833333	0.9954	0.9861	0.9910
Malignant	0.9982	1.0000	0.9991	561	1.			
Normal	0.9905	1.0000	0.9952	416	1.			

### Comparison with state-of-the-art methods

In our proposed method, we have evaluated our model using the Mitscherlich function-based fuzzy ranking-based ensemble approach. Fuzzy ranks are generated for each of the base classifiers, and then these ranks are combined using the ensemble method. The proposed method is compared with the methods found in the literature, and it has been observed that the proposed method achieves the highest accuracy score among others. In comparison, the dataset we have used and compared with others is the same dataset (i.e., the IQ-OTHNCCD lung cancer dataset). Table 8 provides an illustration of the comparative study of our suggested model with others.

**Table 8:** Performance comparison of the proposed ensemble model with state-of-the-art methods on the IQ-OTH/NCCD dataset.

Method	Approach	Acc (%)
Al-Yasriy et al. [57]	AlexNet	95.71
Humayun et al. [67]	VGG16,VGG19 and Xception	98.83
Narin et al. [68]	Alexnet and ResNet50	98.58
Al-Huseiny et al. [69]	Transfer Learning with GoogleNet	94.38
Begum et al. [65]	SMOTE based on 2D-CNN	97.00
Abunajm et al. [66]	CNN	99.45
**Proposed (MENet)**	**An ensemble of CNN models**	**99.54**

Our proposed ensemble approach achieves the best performance accuracy of 99.54% as compared to the other methods or individual classifiers. Out of these, the methods proposed by [57, 65, 66] are based on the deep CNN methods and achieve accuracy of 95.71%, 97.00%, and 99.45%, respectively. [67] have attained 98.83% accuracy by using three transfer learning models, VGG16, VGG19, and Xception, with CNN for the classification of lung cancer on the same dataset. [68, 69] achieve accuracy of 98.58% and 97.38%.

### Comparison with other ensemble techniques

In this section, experimental results are compiled in Table 9 to demonstrate the proposed ensemble scheme’s superiority to well-known traditional ensemble techniques. The ensembles used the identical three base CNN learners, Xception, InceptionResNetV2, and MobileNetV2. The suggested ensemble method performed better than several popularly used ensemble schemes. It is clear from the results that the performance of the weighted average ensemble, which only takes the accuracy metric into account when determining the weights, came the closest to matching the performance of the proposed ensemble technique. In the majority voting-based ensemble, the class that received the highest votes from the base learners is predicted to be the class of the sample.

**Table 9:** Performance comparison of the proposed ensemble method with some commonly used ensemble methods evaluated on the IQ-OTHNCCD dataset. Scores are in %.

Ensemble method	Acc	Pre	Re	F1-score	AUC
Maximum Probability	98.11	98.43	97.79	97.76	97.79
Average Probability	98.82	97.81	97.79	97.78	97.81
Sum Rule	96.55	96.11	96.79	97.47	96.81
Majority Voting	98.11	98.13	98.12	98.10	97.85
Weighted Average	98.92	98.74	98.52	98.20	98.13
**Proposed (MENet)**	**99.54**	**99.62**	**98.61**	**99.10**	**98.88**

### Data visualization

In this section, we take help from two data visualization tools to visually show some results of the proposed method, namely, GradCAM and t-SNE plots.

#### GradCAM analysis

**Fig 13:**
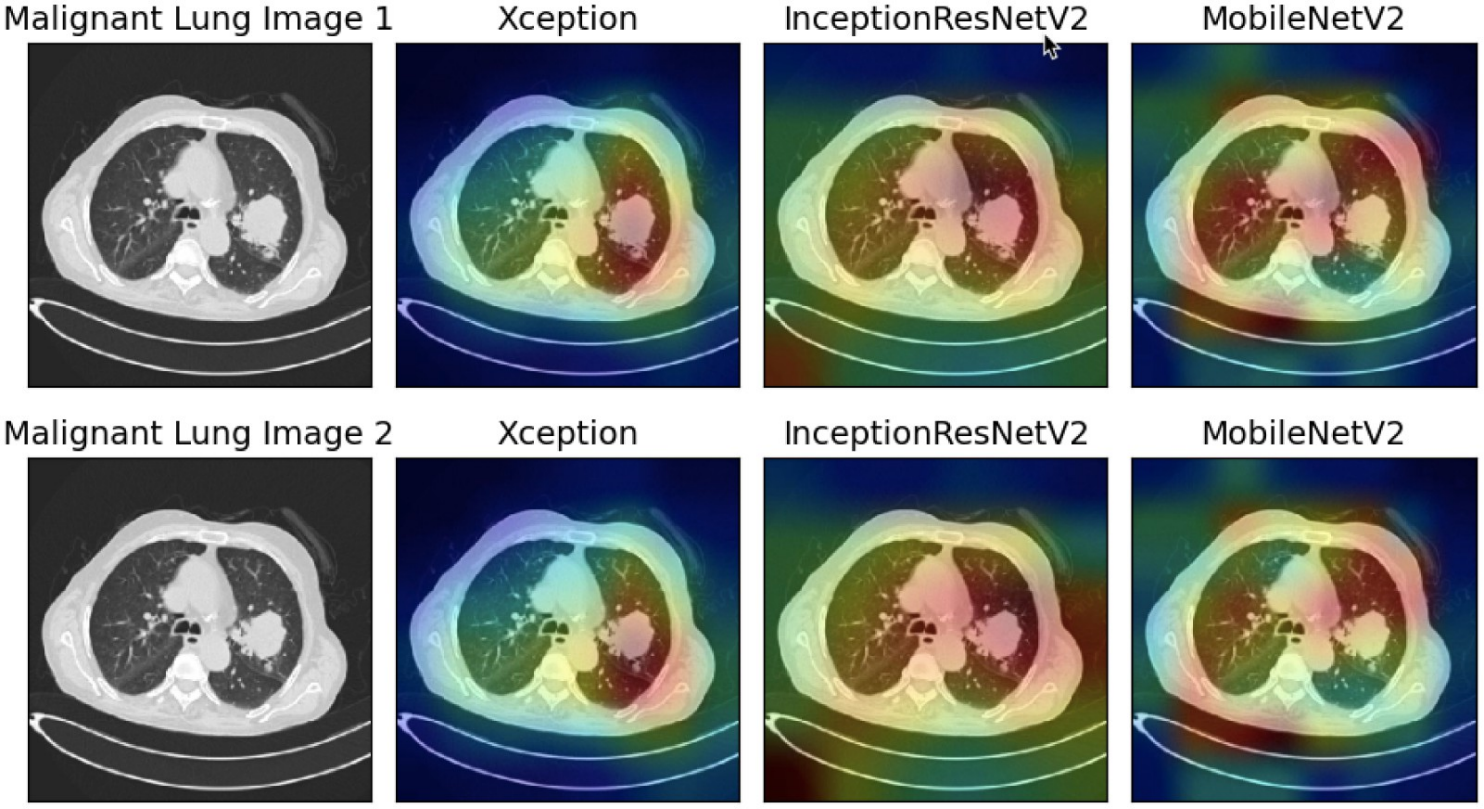
GradCAM visualization of the Malignant lung images taken from the IQ-OTHNCCD dataset with the three base models used to form the ensemble.

**Fig 14:**
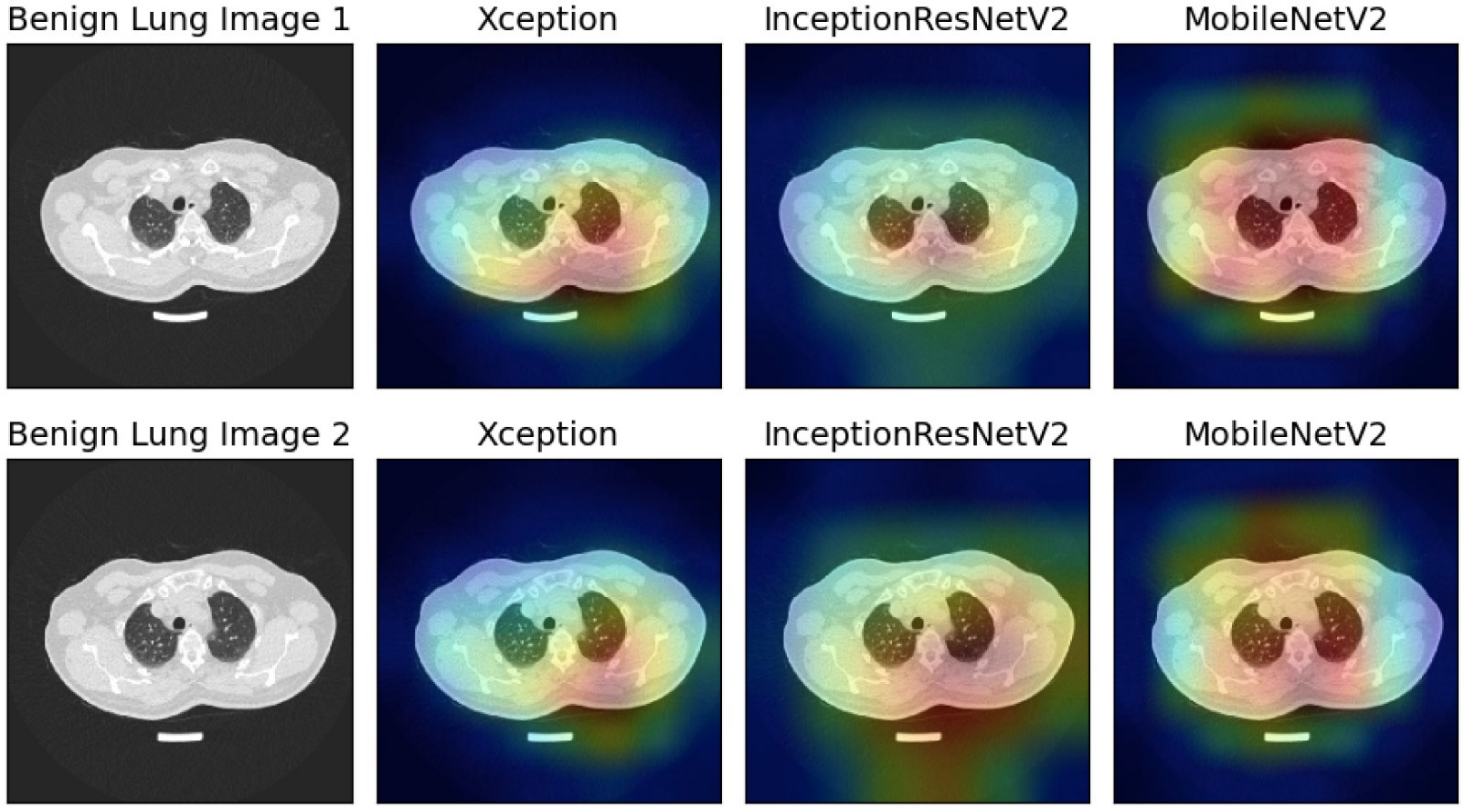
GradCAM visualization of the Benign lung images taken from the IQ-OTHNCCD dataset with the three base models used to form the ensemble.

**Fig 15:**
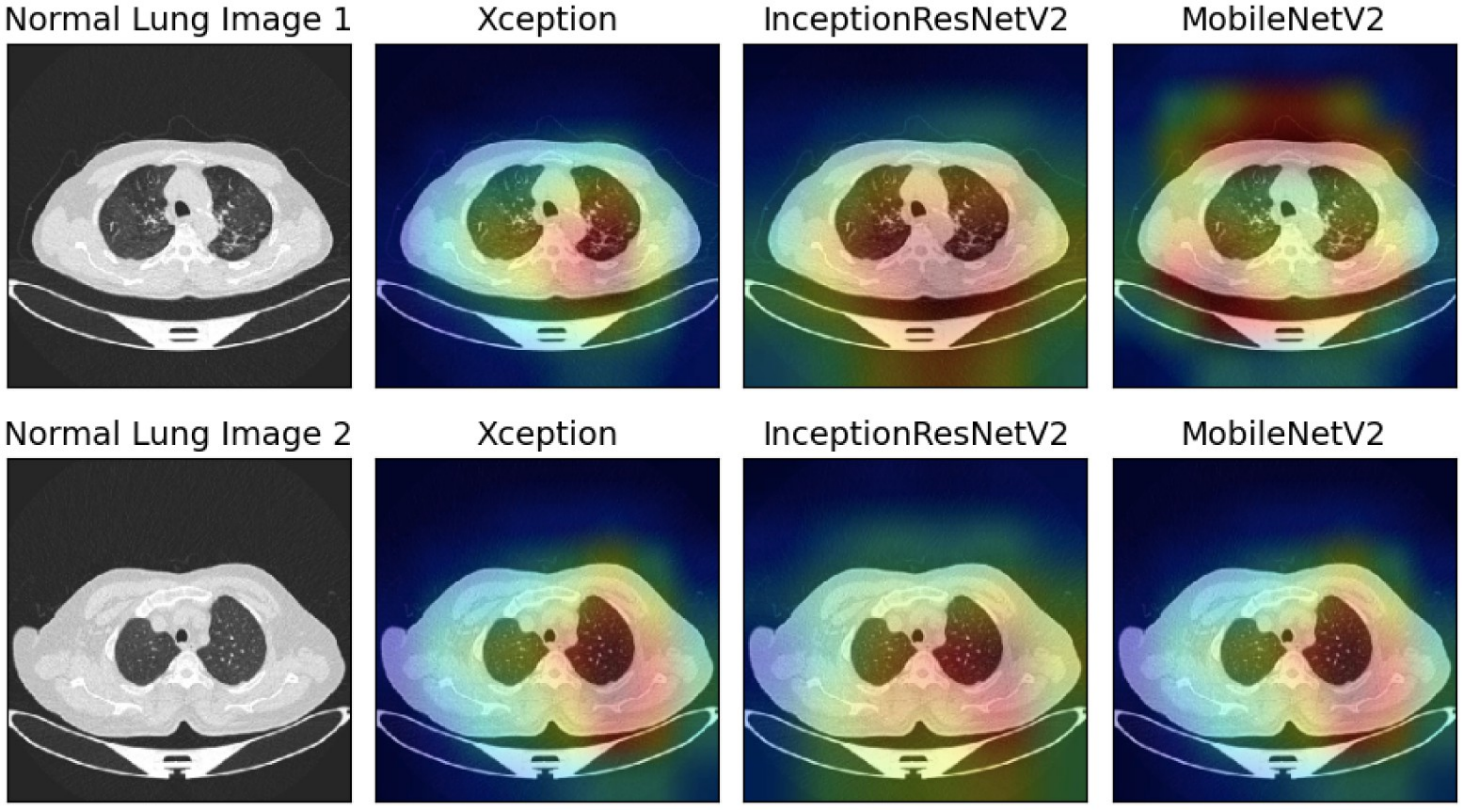
GradCAM visualization of the Normal lung images taken from the IQ-OTHNCCD dataset with the three base models used to form the ensemble.

In this study, we have utilized GradCAM, a technique that creates a gradient-weighted class activation map as proposed by [70], to produce visual explanations of the model predictions. These visualizations help to demonstrate how neural networks arrive at decisions. As shown in Figs 13–15, we have used GradCAM to generate visualizations for the Malignant, Benign, and Normal lung images, respectively from the IQ-OTHNCCD dataset, using the three base models that have been used to construct the ensemble model. It is evident that the various models concentrate on distinct areas of the lung CT scans, thereby suggesting that individual learners capture unique and complementary information that is required to form an ensemble-learning-based model as observed in Fig 13, the Xception model appears to concentrate on the right segment of the lung, the InceptionResNetV2 model on the lung nodules, and the MobileNetV2 model on the corners of the lung. Due to this, the ensemble technique proved to be effective in our present work.

#### t-SNE plots

t-SNE (t-Distributed Stochastic Neighbor Embedding) is a popular dimensionality reduction technique that can be used to visualize high-dimensional data in a lower-dimensional space as proposed by [71]. At first, the algorithm transforms the high-dimensional Euclidean distances among the data points into conditional probability scores that indicate similarities. This is achieved using SNE (Stochastic Neighbor Embedding) on the data points. The conditional probability *P*_*j*|*i*_, which denotes the similarity of data point *x*_*j*_ to data point *x*_*i*_, is defined using the Eq 7.

$$\begin{array}{c} \;P_{i|j}=\frac{exp(\frac{-{\left\|x_{i}-x_{j}\right\|}^{2}}{2\sigma_{i}^{2}})}{\sum_{k\neq i}exp\left(\frac{-{\left\|x_{i}-x_{j}\right\|}^{2}}{2\sigma_{i}^{2}}\right)}\; \end{array}$$ (7)

**Fig 16:**
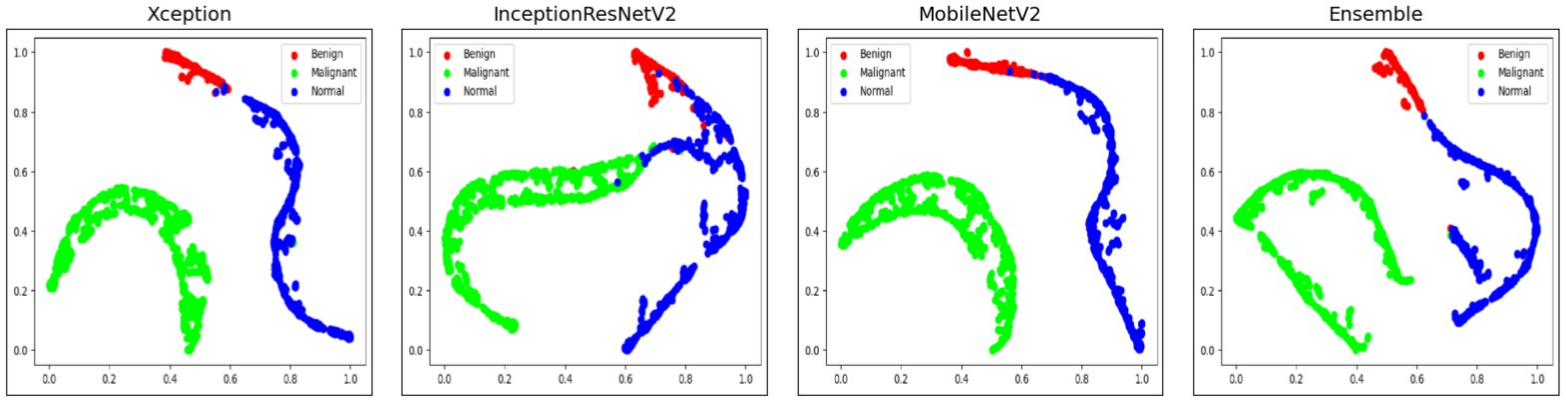
Graphical representation of the t-SNE plots of samples of the IQ-OTHNCCD dataset with the three base models and the final ensemble model.

We observe that the images corresponding to the different stages of lung cancer are clearly separated into different clusters of points. The t-SNE plot visualizations for the Benign, Malignant, and Normal classes of lung nodule CT images from the IQ-OTHNCCD dataset are presented in the first three images of Fig 16. These visualizations have been generated using the three selected base models that form the ensemble. The t-SNE plot of the ensemble model is presented in the fourth image of Fig 16.

### Empirical significance of the ensemble model

**Fig 17:**
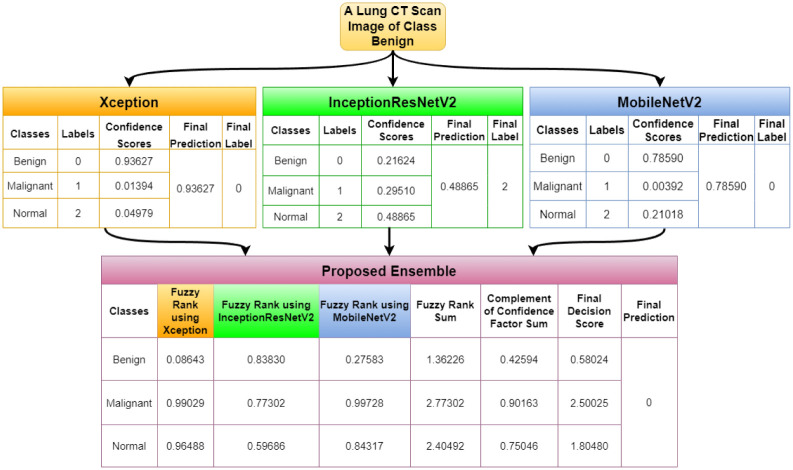
Demonstration for the case when two base models give the correct results, but the third one gives an erroneous result for a Benign image.

**Fig 18:**
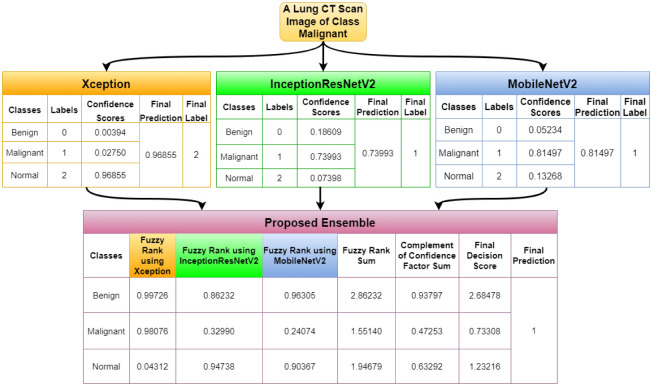
Demonstration for the case when two base models give the correct results, but the third one gives an erroneous result for a Malignant image.

**Fig 19:**
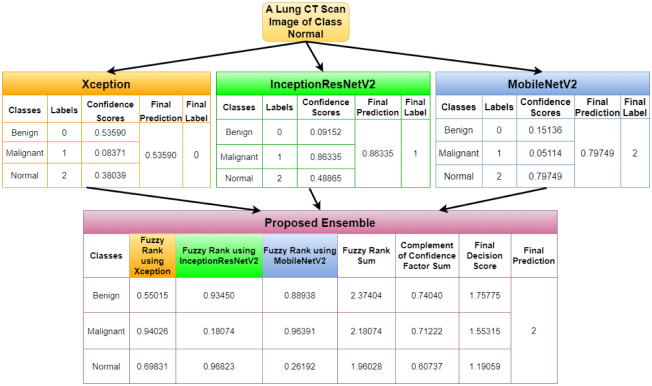
Demonstration for the case when one base model gives the correct results, but the remaining two give erroneous results for a Normal image.

In this section, we report few outlier cases identified during the model testing and analysis. There are cases where the proposed model has performed well, although one or two base classifiers have wrongly classified the sample. Figs 17 and 18 highlight the cases when for a benign image and a malignant image, respectively, two of our base models have given correct answers, and one has not, but our proposed ensemble model is still able to give the correct result. Fig 19 highlights the condition where for a normal image, only one of our base models is able to give the correct result, while the other two could not. In this case, our proposed ensemble method can also give the correct results. These empirical results ensure the effectiveness of the proposed ensemble model in varied scenarios.

### Statistical analysis

We performed McNemar’s statistical test, a non-parametric test, to determine the statistical significance of the results obtained by the proposed ensemble method. The ensemble result is compared against three base models’ results, namely Xception, InceptionResNetV2, and MobileNetV2, on the IQ-OTHNCCD dataset used in this study. The probability scores are used to design the ensemble model. In this case, the null hypothesis is that there is no significant difference in the performance of the three base CNN models in predicting lung cancer outcomes. The results of McNemar’s test are presented in Table 10, and the p-value for the dataset used in this study is found to be less than 0.05 (5%), indicating statistical evidence against the null hypothesis. The statistical test confirms that the proposed ensemble model captures complementary information from the base models and performs better than individual base models. Consequently, none of the contributing base models are statistically equivalent to the ensemble classifier.

**Table 10:** Results of McNemar’s statistical test show that the null hypothesis is rejected for the base models on the IQ-OTHNCCD dataset when compared to the proposed ensemble model, as the p-value is found to be less than 0.05.

Base model	p-value
Xception	0.0014
InceptionResNetV2	0.0317
MobileNetV2	0.0005

## Additional experiments

For further generalization of results, we have executed our proposed model on the LIDC-IDRI [72] dataset, which is also a popular publicly available dataset. The dataset has already been pre-split into train, test and validation sets, each containing images for two classes Benign and Malignant, both of which further contain the CT images of the respective category. Table 11 provides a detailed description of the distribution of the dataset.

**Table 11:** Distribution of images found in the LIDC-IDRI dataset into Benign and Malignant classes.

Dataset	Division	Classes	No. of Images
LIDC-IDRI	Train	Benign	4342
		Malignant	845
	Test	Benign	1340
		Malignant	282
	Validation	Benign	1073
		Malignant	224

**Fig 20:**
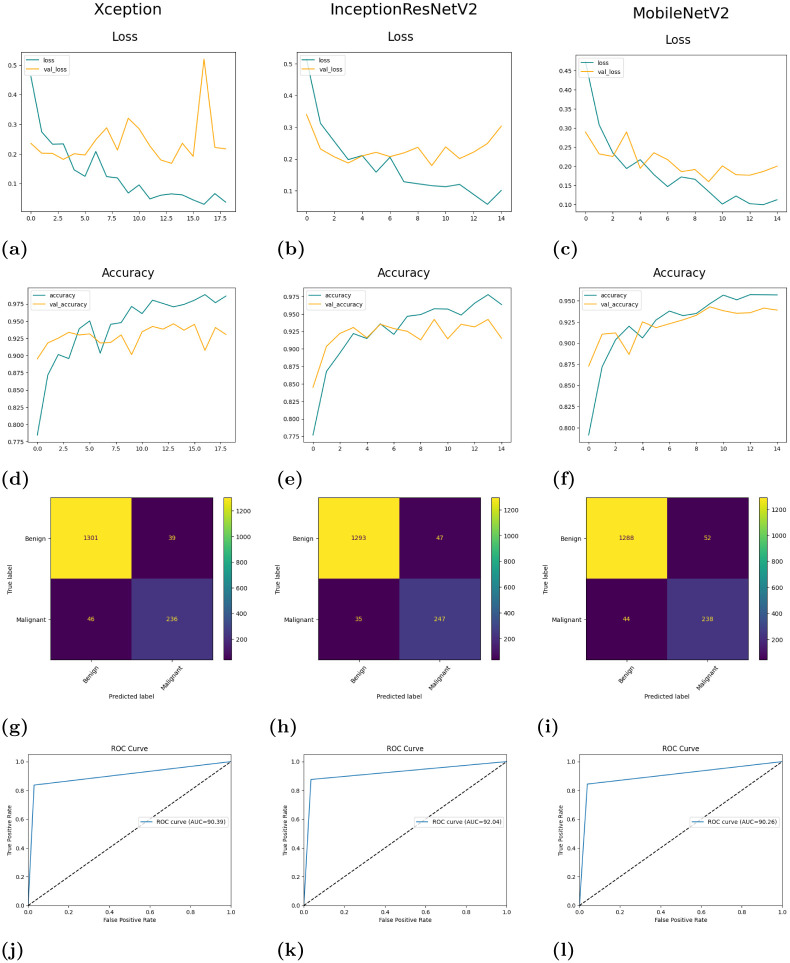
Results obtained from the base models on **LIDC-IDRI dataset**: (a) Loss curve of Xception, (b) Loss curve of InceptionResNetV2, (c) Loss curve of MobileNetV2, (d) Accuracy curve of Xception, (e) Accuracy curve of InceptionResNetV2, (f) Accuracy curve of MobileNetV2, (g) Confusion matrix of Xception, (h) Confusion matrix of InceptionResNetV2, (i) Confusion matrix of MobileNetV2, (j) ROC-AUC curve of Xception, (k) ROC-AUC curve of InceptionResNetV2, (l) ROC-AUC curve of MobileNetV2.

**Fig 21:**
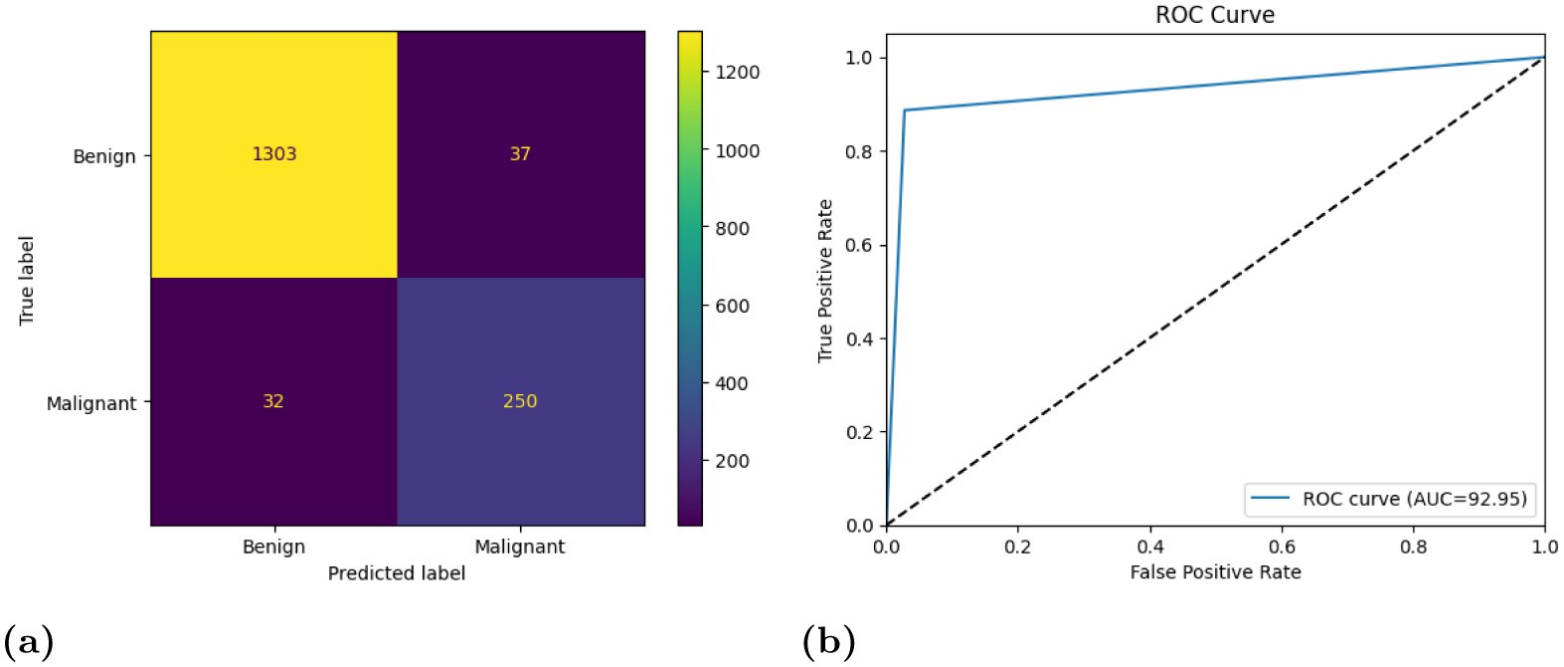
(a) Confusion matrix and (b) ROC-AUC curve, obtained by the proposed ensemble model (MENet) on the LIDC-IDRI dataset.

We use class weights to address the class imbalance in the dataset. To prevent overfitting, we use early stopping while training the model. Fig 20 depicts the loss and accuracy curves of the individual base models along with their confusion matrices and ROC-AUC curves on the test dataset. We can observe from Fig 20 that the loss curves still show a slight tendency of overfitting but overall it shows that our models are able to learn properly with decreasing loss values. The accuracy curves show the high scores obtained by our model. The confusion matrices highlight that although there are some wrong classifications, the ratio of wrong classifications to that of the correct classifications is very low, indicating that these models are reliable for the work. The final ROC curves obtained on the test dataset by each of the models, show that the False Positive rate is significantly low. As the ROC-AUC curves in Fig 20 is shifted towards the top left corner, it indicates the highly efficient ability of the models to classify test images into one of the two classes. Fig 21 show the final ensemble results confusion matrix and ROC-AUC curve.

Each of these base models generates the confidence scores for each class for every single image in the dataset. These confidence scores of all the base models for each image are generated and stored for each individual base models. For the results obtained in Table 12, the three transfer-learned models have been trained for 60 epochs with the Adam optimizer individually.

**Table 12:** Performance measure of each model along with their total number of parameters while experimented on the LIDC-IDRI dataset.

Model	No. of parameters	Acc (%)	Pre (%)	Re (%)
Xception	20,867,627	94.76	91.20	90.39
InceptionResNetV2	54,341,347	94.94	90.69	92.04
MobileNetV2	2,261,827	94.08	89.38	90.26
**Proposed Ensemble**	**77,470,801**	**95.75**	**92.36**	**92.95**

**Fig 22:**
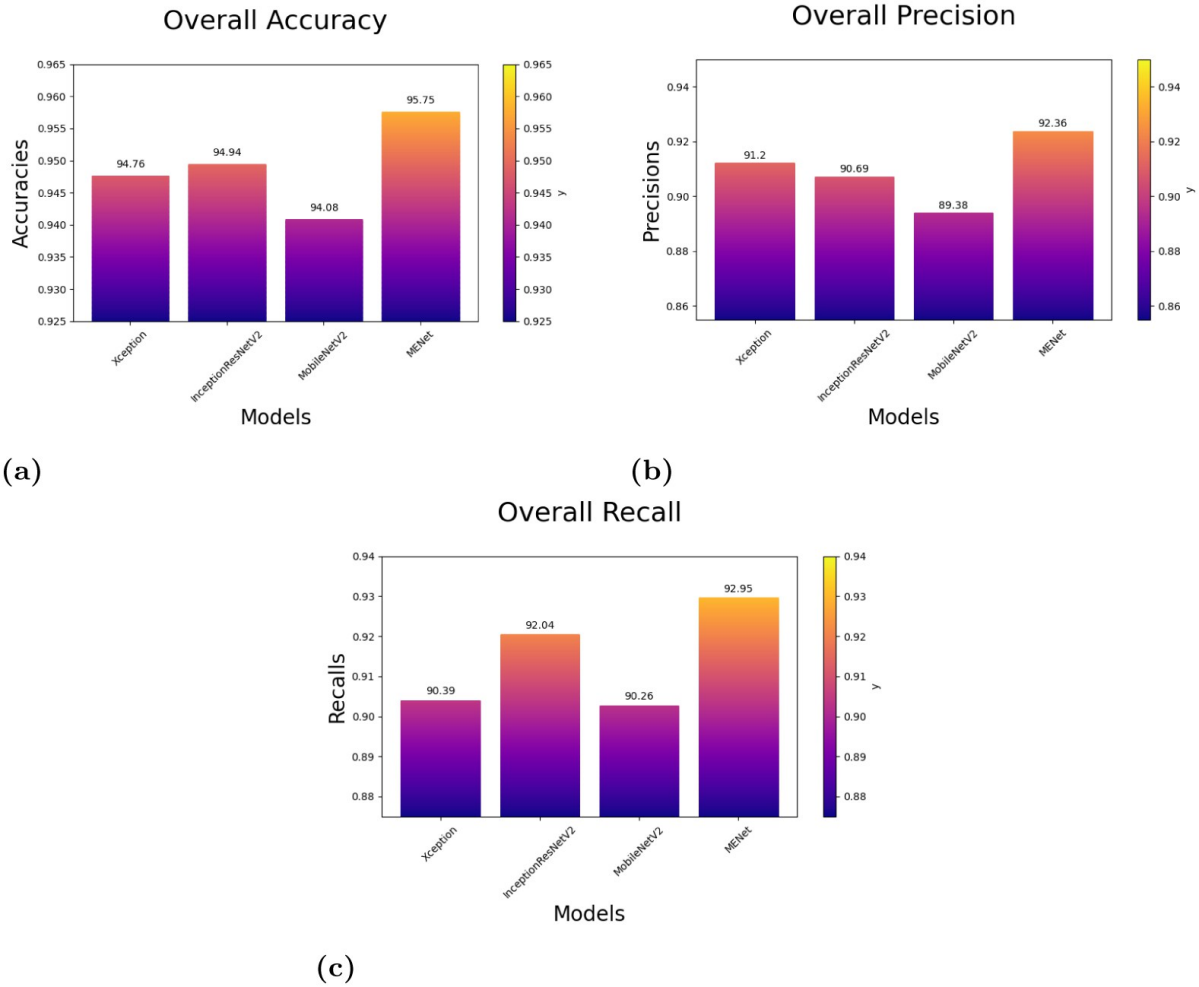
Results of the models depicting their overall accuracy, precision and recall on the **LIDC-IDRI dataset**: (a) Overall accuracy of the models, (b) Overall Precision of the models, (c) Overall Recall of the models.

From Table 13, we can observe that the overall accuracy achieved is 95.75% after combining the results of the three base classifiers. The class-wise results have also been given in the Table 13. All these classification scores indicate that the model is highly reliable. Fig 22 provides a detailed pictorial representation of the performances of each base model and the final ensemble method on the LIDC-IDRI dataset.

**Table 13:** Class-wise and overall results obtained by the proposed ensemble model on the LIDC-IDRI dataset.

Class	Pre	Re	F1	Support	Acc	Overall Acc	Overall Re	Overall F1
Benign	0.9760	0.9724	0.9742	1340	0.9724	0.9575	0.9295	0.9265
Malignant	0.8711	0.8865	0.8787	282	0.8865			

### Comparison with past methods

The proposed method is compared with the methods found in the literature review, and it has been observed that the proposed method achieves the highest accuracy score among others. In comparison, the dataset we have used and compared with others is the same dataset (i.e., the LIDC-IDRI lung cancer dataset). Table 14 provides an illustration of the comparative study of our suggested model with others.

**Table 14:** Performance comparison of the proposed ensemble model with state-of-the-art methods for the LIDC-IDRI dataset.

Work Ref.	Method	Acc (%)
Zhao et al. [32]	Forward and Backward GAN and Multi-scale VGG16	95.20
Bhatia et al. [34]	Deep Residual Learning	84.00
Shaffie et al. [24]	Resolved Ambiguity Local Binary Pattern	94.90
Xie et al. [73]	Semi-supervised Adversarial Classification mode	92.50
Baoxian et al. [74]	Novel DCNN	94.00
Hanliang et al. [75]	Convolutional block attention module	90.70
Shafi et al. [28]	Deep Learning-Assisted SVM-Based Model	94.00
AR et al. [76]	LCD-CapsNet	94.00
**Proposed (MENet)**	**An ensemble of three CNN models**	**95.75**

## Conclusion and future scope

In recent times, it has been observed that CAD systems can help diagnostic precision and decrease the likelihood of human errors by automating the diagnosis process. In the present work, we have designed an ensemble model, called MENet, using three transfer learning-based CNN models, namely Xception, InceptionResNetV2, and MobileNetV2, to enhance the accuracy of a lung cancer prediction model. In doing so, we have used a fuzzy ranking-based approach, which considers the uncertainty of each classifier’s predictions and assigns different levels of importance to each classifier. The fuzzy ranking system is designed based on the Mitscherlich function, which combines the outputs of the base classifiers to form a final prediction model that is more accurate than each classifier’s individual prediction ability. The proposed method has been evaluated on the two lung CT scan datasets, namely IQ-OTHNCCD and LIDC-IDRI, and the obtained results are better than many recently proposed methods.

However, there are some false positives and false negatives, and these are significant challenges in the medical field as it would directly affect the treatment of patients. Hence, in the future, we need to reduce such errors. For this purpose, we may apply some attention mechanisms to the base CNN models that might help to generate better feature maps by focusing on the important regions, which, in turn, might produce a better prediction model. In the future, we would like to explore some lightweight CNN models to make the overall model to be useful in practical cases. Moreover, we recognize the importance of thoroughly analyzing and mitigating potential problems associated with our proposed method. Future work will involve a comprehensive examination of challenges, ranging from interpretability issues to scalability concerns. Developing strategies to address these challenges will contribute to the robustness and dependability of the MENet across diverse medical settings. Considering the evolving nature of medical datasets, the model’s adaptability to new information and potential concept drift can also be assessed as future work.

## Supporting information

S1 Appendix Dry run of the entire proposed method. (PDF)
