# Supplementary material for: MENet: A Mitscherlich function based ensemble of CNN models to classify lung cancer using CT scans
Source: PLoS One. 2024 Mar 11;19(3):e0298527. doi: 10.1371/journal.pone.0298527 (PMC10927148; doi:10.1371/journal.pone.0298527)
Supplement: S1 Appendix — (PDF) [file pone.0298527.s001.pdf]

A Dry Run of the entire proposed method

In this section, we provide a dry-run demonstration of the proposed fuzzy-ranking-based ensemble method, called MENet, applied for lung cancer detection using CT scans. This dry-run would help the common readers to understand better. This dry run's steps include generating confidence scores, merging these scores using the proposed Mitscherlich function-based ensemble procedure, and obtaining the final prediction on test data.

The confidence or probability scores of the training and testing images are generated by individual base classifiers and stored in their individual CSV files.

Table 6 in the main paper shows the results of the selected base models.

Table A1. Sample contents of the CSV file for the three base classifiers showing the confidence scores of each class: Xception (Table A1a), InceptionResNetV2 (Table A1b) and MobileNetV2 (Table A1c)

Benign	Malignant	Normal
0.8627	0.0002	0.1371
0.5998	0.2816	0.1185
0.8915	0.0071	0.1013
0.7174	0.0036	0.2789
0.9304	0.0012	0.0684
0.9363	0.0139	0.0498

(a) Xception

Benign	Malignant	Normal
0.8456	3.81E-07	0.1544
0.7019	0.00021	0.2978
0.5832985	2.85E-06	0.4167
0.7554	0.1430	0.1016
0.6337	6.50E-06	0.3663
0.6286	0.2517	0.1197

(b) InceptionResNetV2

Benign	Malignant	Normal
0.9112	1.45E-06	0.0888
0.9123	2.41E-06	0.0876
0.9257	1.32E-06	0.0743
0.7389	0.0793	0.1818
0.9659	3.36E-07	0.0341
0.7188	0.0197	0.2616

(c) MobileNetV2

In Table A1, columns represent classes (Benign, Malignant, Normal), and rows represent images in the dataset. Here the tables show results for six randomly chosen samples.

Then using the Mitscherlich function in Equation 2 (in the main paper), we generate the fuzzy ranks for all the samples that belong to various classes.

$$A_1 = \begin{pmatrix} [0.08036 & 0.99997 & 0.95817] \\ [0.18151 & 0.99986 & 0.90033] \\ [0.09625 & 0.99996 & 0.94948] \\ [0.96089 & 0.99964 & 0.07595] \\ [0.88624 & 0.99995 & 0.20436] \\ [0.97705 & 0.99958 & 0.04569] \end{pmatrix} \tag{8}$$

$$A_2 = \begin{pmatrix} [0.20297 & 0.99999 & 0.88705] \\ [0.37327 & 0.99985 & 0.77073] \\ [0.50173 & 0.99999 & 0.66513] \\ [0.87021 & 0.99563 & 0.23746] \\ [0.94939 & 0.99989 & 0.09654] \\ [0.88377 & 0.99950 & 0.20914] \end{pmatrix} \tag{9}$$

$$A_3 = \begin{pmatrix} [0.11936 & 0.99999 & 0.93653] \\ [0.11789 & 0.99999 & 0.93736] \\ [0.10038 & 0.99999 & 0.94716] \\ [0.99048 & 0.99923 & 0.02038] \\ [0.99239 & 0.99996 & 0.01520] \\ [0.91669 & 0.99823 & 0.15706] \end{pmatrix} \quad (10)$$

The matrices $A_1,\ A_2\ and\ A_3$ in Equation 8, Equation 9 and Equation 10 show sample fuzzy ranks generated using Equation 2 (in the main paper) for Xception, InceptionResNetV2 and MobileNetV2 base classifier, respectively. Each column represents a different class (Benign, Malignant, and Normal), whereas each row represents an image from the dataset.

Now with the help of Equation 3 (in the main paper) and Equation 4 (in the main paper), we generate the fuzzy rank sum and the complement of confidence factor sum as shown in Equation 11 by matrix B and Equation 12 by matrix C.

$$B = \begin{pmatrix} [0.40269 & 3. & 2.78176] \\ [0.67267 & 3. & 2.60843] \\ [0.69836 & 3. & 2.56177] \\ [2.82158 & 3. & 0.33379] \\ [2.82802 & 3. & 0.31609] \\ [2.77752 & 3. & 0.41189] \end{pmatrix} \quad (11)$$

$$C = \begin{pmatrix} [0.10078 & 1. & 0.89924] \\ [0.17431 & 1. & 0.82583] \\ [0.18738 & 1. & 0.81264] \\ [0.91831 & 1. & 0.08432] \\ [0.92080 & 1. & 0.07929] \\ [0.89773 & 1. & 0.10356] \end{pmatrix} \quad (12)$$

Both in matrix B in Equation 11 and matrix C in Equation 12, columns, as usual, represent classes in the order Benign, Malignant, and Normal, whereas rows represent each CT scan image in the dataset. Matrix B reflects the fuzzy rank sum, which provide valuable insights into the relative importance or ranking of data points, while matrix C represents the complement of confidence factor, which indicate the degree of uncertainty or confidence in the data. By using both matrices, we are able to leverage a broader range of information to make more informed decisions.

To obtain the final decision score, we perform element-wise multiplication between the fuzzy rank sum matrix B and the complement of confidence factor sum matrix C that will be used to generate the final predictions of our ensemble approach. We use the Equation 5 to get the result represented by matrix D in Equation 13.

$$D = \begin{pmatrix} [0.04058 & 3. & 2.50147] \\ [0.11726 & 3. & 2.15411] \\ [0.13086 & 3. & 2.08179] \\ [2.59109 & 3. & 0.02815] \\ [2.60404 & 3. & 0.02506] \\ [2.49347 & 3. & 0.04266] \end{pmatrix} \quad (13)$$

In the end, to get the final prediction of the proposed MENet model, we use Equation 6. Hence, based on it, the final prediction for:

$$
\begin{aligned}
image_1 &= \min(0.04058,\ 3.,\ 2.50147) = 0.04058 = Benign \\
image_2 &= \min(0.11726,\ 3.,\ 2.15411) = 0.11726 = Benign \\
image_3 &= \min(0.13086,\ 3.,\ 2.08179) = 0.13086 = Benign \\
image_4 &= \min(2.5911,\ 3.,\ 0.0282) = 0.0282 = Normal \\
image_5 &= \min(2.6040,\ 3.,\ 0.0251) = 0.0251 = Normal \\
image_6 &= \min(2.4935,\ 3.,\ 0.0427) = 0.0427 = Normal
\end{aligned}
$$

This is how our proposed ensemble model, called MENet, classifies images into Benign, Malignant, and Normal categories.
